# Alkaline air: changing perspectives on nitrogen and air pollution in an ammonia-rich world

**DOI:** 10.1098/rsta.2019.0315

**Published:** 2020-09-28

**Authors:** Mark A. Sutton, Netty van Dijk, Peter E. Levy, Matthew R. Jones, Ian D. Leith, Lucy J. Sheppard, Sarah Leeson, Y. Sim Tang, Amy Stephens, Christine F. Braban, Ulrike Dragosits, Clare M. Howard, Massimo Vieno, David Fowler, Paul Corbett, Mohd Irfan Naikoo, Silvana Munzi, Christopher J. Ellis, Sudipto Chatterjee, Claudia E. Steadman, Andrea Móring, Patricia A. Wolseley

**Affiliations:** 1 UK Centre for Ecology & Hydrology, Edinburgh Research Station, Bush Estate, Penicuik, UK; 2 Northern Ireland Environment Agency, Belfast, UK; 3 Department of Botany, Aligarh Muslim University (AMU), Aligarh, India; 4 Centro Interuniversitário de História das Ciências e da Tecnologia, Faculdade de Ciências, Lisbon, Portugal; 5 Centre for Ecology, Evolution and Environmental Changes, Faculdade de Ciências, Lisbon, Portugal; 6 Royal Botanic Garden Edinburgh (RBGE), Edinburgh, UK; 7 Department of Natural Resources, TERI School of Advanced Studies (TERISAS), New Delhi, India; 8 School of Geosciences, University of Edinburgh, Edinburgh, UK; 9 Natural History Museum, Cromwell Road, London, UK

**Keywords:** alkaline air, nitrogen, *nūshādir*, lichens, ecosystem recovery, circular economy

## Abstract

Ammonia and ammonium have received less attention than other forms of air pollution, with limited progress in controlling emissions at UK, European and global scales. By contrast, these compounds have been of significant past interest to science and society, the recollection of which can inform future strategies. Sal ammoniac (*nūshādir*, *nao sha*) is found to have been extremely valuable in long-distance trade (*ca* AD 600–1150) from Egypt and China, where 6–8 kg N could purchase a human life, while air pollution associated with *nūshādir* collection was attributed to this nitrogen form. Ammonia was one of the keys to alchemy—seen as an early experimental mesocosm to understand the world—and later became of interest as ‘alkaline air’ within the eighteenth century development of pneumatic chemistry. The same economic, chemical and environmental properties are found to make ammonia and ammonium of huge relevance today. Successful control of acidifying SO_2_ and NO*_x_* emissions leaves atmospheric NH_3_ in excess in many areas, contributing to particulate matter (PM_2.5_) formation, while leading to a new significance of alkaline air, with adverse impacts on natural ecosystems. Investigations of epiphytic lichens and bog ecosystems show how the alkalinity effect of NH_3_ may explain its having three to five times the adverse effect of ammonium and nitrate, respectively. It is concluded that future air pollution policy should no longer neglect ammonia. Progress is likely to be mobilized by emphasizing the lost economic value of global N emissions ($200 billion yr^−1^), as part of developing the circular economy for sustainable nitrogen management.

This article is part of a discussion meeting issue ‘Air quality, past present and future’.

## 1. Introduction

Over recent decades ammonia (NH_3_) has often seemed like the Cinderella of air pollution, as it has been given much less attention than other pollutants, such as sulfur dioxide (SO_2_), nitrogen oxides (NO*_x_*), ozone (O_3_) and particulate matter (PM). In the 1980s, research focused on ‘acid rain’, especially in the light of SO_2_ and NO*_x_* emissions [1–3] with only a few researchers at that time examining the possible effects of NH_3_ and ammonium $\left(\text{N}{{\text{H}}_{4}}^{+}\right)$ on the environment, including threats to soils, biodiversity and forest health [4–6]. The same can be said for European air pollution policy, with successive international protocols on SO_2_ and NO*_x_* emissions [7,8], preceding the multi-pollutant, multi-effect Gothenburg Protocol [9], which included NH_3_ for the first time. Even then, the commitments for NH_3_ were much less ambitious than for other air pollutants, requiring that little action be taken by most countries. The situation is similar with the 2020 ceilings of the revised Gothenburg Protocol of 2012. With insufficient measures implemented, several countries are unlikely to meet their legally binding NH_3_ ceilings for 2020, while overall Europe-wide NH_3_ emissions have actually been increasing since 2013 [10]. The barriers appear to be primarily political, as The Netherlands and Denmark have shown that it is possible to reduce NH_3_ emissions substantially.

With this perspective in mind, it is appropriate to take stock of what ammonia has meant to people in the past, what it means today, and what it might mean in the future. We rapidly discover that NH_3_ and $\text{N}{{\text{H}}_{4}}^{+}$ were historically far from insignificant, fulfilling several important roles. Whereas recent efforts have focused on reducing NH_3_ emissions from agriculture, with the main sources being livestock excreta and fertilizers, the historical picture helps to raise awareness of the multi-dimensional relevance of ammonia for environment and society.

Considering the present, across much of Europe and North America we now inherit a world where substantial emission controls have already been achieved for SO_2_ and NO*_x_*. We consider in detail the implications of the changed ratio of NH_3_ to the acid gases, especially for some of the most sensitive ecological receptors. Instead of acid rain, we now face challenges from ‘alkaline air’, which was the original name given by Joseph Priestley [11] for gaseous ammonia. Today, we may also define alkaline air more generally as air where alkaline gases (primarily NH_3_, but in principle also including volatile amines) dominate over those that are acidic in nature.

Finally, we consider what might be expected for the future. What are the implications of current legislation, of the slightly more ambitious emission reductions for 2030 under the revised EU National Emissions Ceilings Directive (2016/2284/EU)? We conclude by placing NH_3_ mitigation in the context of the circular economy for nitrogen and United Nations actions on nitrogen to help meet multiple Sustainable Development Goals (SDGs).

In the following sections, we show how a broad approach linking past, present and future could help raise awareness about the importance of ammonia and nitrogen as a contribution to catalysing action on the SDGs. We juxtapose the historical value of ammonium in international trade and alchemy with current development of the nitrogen circular economy. The analysis is underpinned with a more detailed examination of ecological datasets for epiphytic lichens and bog ecosystems which together emphasize the emerging importance of alkaline air.

## 2. Ammonia in the past and implications for the present

While the popular historical narrative ascribes the discovery of ammonia to Priestley [11], his achievement needs to be set in the context of at least two millennia of human exploration and investigation into ammonia and ammonium.

### (a) Ammonia in ancient times

**Figure 1.**
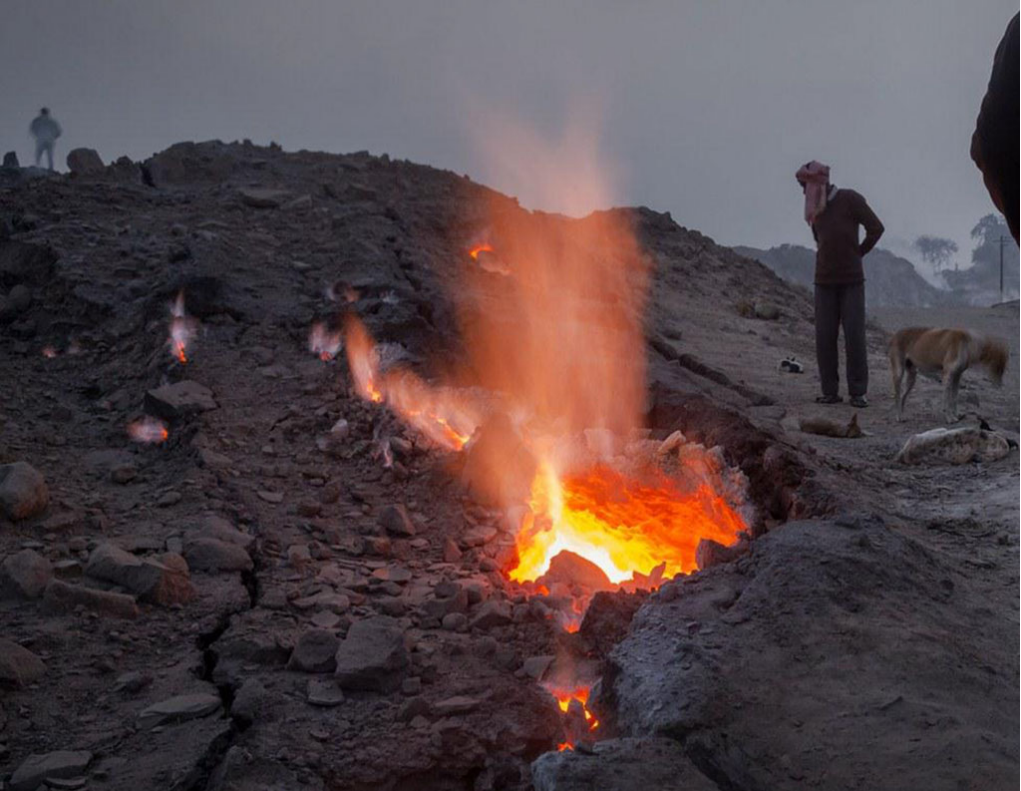
Fire cave at Jharia, India, where spontaneous combustion of surface coal deposits has resulted in burning at this location for over a century. Sites such as this across Central Asia, together with volcanic fumaroles, represent the earliest recorded sources of traded ammonium salts (Photo © Johnny Haglund). (Online version in colour.)

By the start of the Tang Dynasty (AD 618–907), ammonium salts for use in metallurgy, medicine and food were already being traded as a luxury product along the Silk Road in Central Asia [12]. Spontaneous combustion of near-surface coal deposits explains the development of fire caves, some of which burn for hundreds of years. Nitrogen (N) in the burnt coal volatilizes as NH_3_, reacting with co-emitted hydrochloric acid (HCl), sulfuric acid (H_2_SO_4_) and nitric acid (HNO_3_) to form a mix of ammonium chloride, sulfate and nitrate salts [13]. Ammonium chloride tends to dominate in the collected sublimate (also known as *nūshādir*, *nao sha*, sal ammoniac, the Eagle (*nasr*) and a wealth of other names), presumably because it is more volatile than ammonium sulfate, while ammonium nitrate formation may be limited by low HNO_3_ concentrations relative to NO*_x_* (ammonium nitrate is also decomposed to N_2_, N_2_O and water at high temperatures). Along with many other point sources, NH_3_ emissions from such fire caves can now be detected from space [14], such as at Jharia in India [10] (figure 1).

The historical collection of the sal ammoniac sublimate around the cooler edge of fire caves, as well as from a range of volcanic fumaroles (from Etna in Sicily to Mount Damavand in Iran), allowed it to become a key commodity of long-distance trade up to the early nineteenth century [12]. The importance and the stability of the sal ammoniac market can be illustrated by comparing prices from AD 620 (Central Asia) with those from AD 1000–1140 (Mediterranean trade) as shown in table 1. The estimates for Central Asia are based on transactions recorded in tax records discovered near Turfan, in present-day Xinjiang province of China [15]. These values are compared with documentary records recovered from a *geniza* or document repository, as uniquely preserved in Cairo [16].

**Table 1.** Comparison of sal ammoniac prices with spice, silk and slaves for Central Asian and Mediterranean trade during the seventh and eleventh to twelveth centuries. For calculations, see electronic supplementary material, §1.

		prices in *nuqra* dirhams (pure silver dirhams, d)^a^				
location (main trade locations)	date	sal ammoniac (d/kg)	spice (d/kg)	silk (d/kg)	slaves (d/slave)	N cost of a human (kg N/slave)^b^
Turfan (China, Central Asia)	*ca* 620	6^c^	5	17	120	6
Egypt (Sicily, Tunisia)	1000–1140	7.6 (5.9–10.6)^d^	5	59	243 (208–278)^d^	8.4 (5.7–11.1)^d^

^a^Derived from data for Turfan [15] and Egypt [16].

^b^Converted based on N content of sal ammoniac of 26.2%.

^c^The estimates for Turfan draw on six transactions for sal ammoniac, of which one includes the amount of tax paid, with a second combined transaction of sal ammoniac and spice that agrees within 10%.

^d^95% confidence limits with *n* = 12 and 19 for sal ammoniac and slave price, respectively.

Table 1 shows impressive similarity for the prices of sal ammoniac and spice from these independent datasets, while the price of silk and slaves had increased substantially in Cairo compared with Central Asia. With today's perspective, it is shocking to note that just 6–8 kg N would purchase a human being. This reflects both a high price of nitrogen and a low value of human life compared with the present. Relative to changing gold and silver prices (electronic supplementary material, §2), N compounds are today around three orders of magnitude cheaper, with prices decreasing rapidly during the twentieth century as large-scale manufacture, mainly through the Haber–Bosch Process [17], has increased their availability.

The burning coal caves of Central Asia also provide the first recorded example of ammoniacal air pollution. It appears that locals would encourage the natural coal burning specifically to harvest sal ammoniac, as recorded by ibn-Hauqal:

Over the spot whence the vapour issues, they have erected a house, the doors and windows of which are kept so closely shut and plastered over with clay that none of the vapour can escape. On the upper part of this house the *nūshādir* rests. When the doors are to be opened, a swiftly running man is chosen, who having his body covered over with clay, opens the door; takes as much as he can of the *nūshādir*, and runs off; if he should delay, he would be burnt (translated by Ouseley [18], p. 264, who renders *nūshādir* as ‘copperas’).

Further details of the pollution threat are given by al-Mas'ūdī:

Travellers in summer take their road from Khorāsān to China by this mountain; for there is a valley through it, which is forty or fifty miles long. At the entrance of the valley wait some men who offer themselves to carry the baggage, if they are well paid. They use sticks to drive the passengers on their journey; for any stoppage or rest would be fatal to the traveller, in consequence of the irritation which the ammoniacal vapours of this valley produce on the brain, and on account of the heat. The way becomes more and more narrow till the travellers come to the end of their perilous passage. Here are pits with water, in which they throw themselves, to obtain relief … When travellers arrive in the Chinese territories, they are beaten as in passing (to counteract the congestion of blood in the brain) (translated by Sprenger [19], pp. 359–360).

Caution is needed with regard to the comment of al-Mas’ūdī about effects of *nūshādir* on the brain. This may reflect the fact that the Chinese term, *nao sha*, includes a component referring to the brain, so that *nao sha* was sometimes termed brain salt (see [20], pp. 446–447), for which there are several possible explanations.

### (b) Early ammonia science and philosophy

While the above examples illustrate the historic importance of ammonium in trade and air pollution, these were probably not the earliest applications. Pliny the Elder (*Natural History* 28: 19, 149) was already familiar with use of the fumes of deer horn and hair to make people breathe naturally when choking with hysteria. This use is directly analogous to the eighteenth century popularity of ammonium carbonate as ‘smelling salts’; these liberate gaseous NH_3_, which acts as a vasodilator in the airways. Ammonia and ammonium were also known in scientific circles, if not always openly. In particular, they were at the heart of alchemy, well-known as a ‘reserved’ science (i.e. unspoken, secret, limited to the few), making it extremely difficult to trace how they were used.

One of the most clear alchemical writers on *nūshādir* was the Persian physician al-Rāzī. It has often been stated that earlier Greek alchemy used exclusively metals and other minerals, while Islamic alchemy introduced the use of organic materials (e.g. [20], p. 435, [21]). The following illustrates the methods of al-Rāzī:

Take of black cleaned hair, distil its water and oil and calcine its residue according to what is [explained] further above, and put away each part of it separately … Then tie it [the solidified oil] up in a linen cloth and hang it into distilled urine in a clay container on the hook of the blind [cucurbit]. Place it on a small oven under which burns a fire of a lamp. Leave it for 24 h, that the urine becomes red. Then pour it off and renew the urine. Repeat this operation until all the colour is extracted. Then gather all and distil. Distil white urine, but its redness remains. Then mix that what remained from the oil in a batch with the distilled juice of a lemon and treat it with the urine with the help of the operation…

Then convert it into a hard state in a blind [cucurbit]; it solidifies it into white *nuqra* like crystal … But if you want, that it [transmutes] into the red [into gold], thus put in it before it solidifies, the red [residue] … it solidifies, transmuting into red *nuqra*, a dirham of which transmutes 1800 dirham of any metal whichever you want into pure gold (trans. by G. Fischer from [22], pp. 109–110).

Special caution is needed here, as al-Rāzī uses so-called ‘cover names’ (*Decknamen*), referring to the ammoniacal distillates as ‘urine’ (because of how it comes out of the alembic) or ‘lemon juice’ (because of its sharpness). Considering these processes, the Arab/Persian alchemist Jābir refers to alchemy as a mesocosm or middle-world, which links understanding of the macrocosm (universe) with the microcosm (humans) (cf. [23], p. 74). In experiments like this, ammonia and ammonium were key to early experimental philosophy. As to the gold, even more caution is needed. In the margin of one manuscript, one of al-Rāzī's readers commented: ‘Truly I have looked into this book … Do not occupy yourself with them [the essences of Arsenic and Sulphur] unless you already know the secret of the process … Only if you know the secret, God willing, will you accomplish the work’ (translated by Heym [24], p. 191).

In fact, it looks likely that earlier Greek alchemists were already familiar with ammonia and ammonium salts. For example, characteristic steps from the al-Rāzī process given above can be found in writings of the Greek alchemist Zosimus (e.g. [25], pp. 30–33; [26], pp. 486–492) and in those attributed to Democritus (e.g. [27], p. S91; electronic supplementary material, §3). There is also a question about the oldest name for sal ammoniac. The term *nūshādir* appears earliest in its Chinese rendering as *nao sha*, but has a well-established Iranian etymology, meaning ‘immortal fire’ [12]. It is a name that matches just as well to the macrocosmic fire caves as to the processing of ‘elements’ in the mesocosmic analysis of earlier Greek alchemy, leaving open the question of its origin.

Obscure as these beginnings may seem, they form the foundations on which modern science was built. This is no more apparent than with Isaac Newton, who experimented and wrote extensively on alchemy, but deliberately kept his findings secret (e.g. [28], p. 159) and encouraged others to do so. Newton thus wrote to Henry Oldenburg, the Secretary of the Royal Society, encouraging Robert Boyle not to reveal alchemical secrets:

[It] may possibly be an inlet to something more noble, not to be communicated without immense dammage to ye world if there should be any verity in ye Hermetick writers, therefore I question not but that ye great wisdom of ye noble Authour [Boyle] will sway him to high silence till he shall be resolved of what consequence ye thing may be … there being other things beside ye transmutation of metals … which none but they [the alchemists] understand … but pray keep this letter private to your self [29].

The message was the traditional one of many alchemists over the centuries: not to reveal the secrets of alchemy, which could otherwise lead to the destruction of society (cf. al-Jildakī [30], p. 49). While Boyle may have engaged in the practice of advertising secrecy [31], Newton appears to have recognized the ethical dilemma concerning open explanation of alchemy.

### (c) The discovery of ‘alkaline air’

Ultimately, the scientific community turned away from the secrecy of alchemy, pushing towards openness of scientific publication for practical benefit. As the experimentalist Stephen Hales wrote in the year that Newton died:

If those who unhappily spent their time and substance in search after an imaginary production, that was to reduce all things to gold, had, instead of that fruitless pursuit, bestowed their labour in searching after this much neglected volatile Hermes, who has so often escaped thro’ their burst receivers, in the disguise of a subtile spirit, a mere explosive matter; they would then instead of reaping vanity, have found their researches rewarded with very considerable and useful discoveries ([32], p. 180).

Hales’ experiments were to be decisive as a prelude to the scientific discovery of ammonia. His work introduced the idea of ‘pneumatic chemistry’, distilling all sorts of products and then collecting the resulting gases in an inverted vessel over a trough of water. In the case of ammonia distilled from blood or harts-horn, this first filled the vessel, but then gradually dissolved in the water, leaving Hales with no ammonia to collect (e.g. [32], p. 95, Experiment XLIX). Continuing these kinds of experiments 50 years later, Joseph Priestly instead filled his pneumatic trough with mercury in which the ammonia would not dissolve. This enabled him to isolate and characterize pure ammonia gas [11]. Priestley's first report was in a private letter to Benjamin Franklin in September 1773, later presenting his findings to the Royal Society ([33], pp. 93–99).

It was only in the 1790s that Priestley's alkaline air started to become known as ‘ammonia pura’, given its relationship to sal ammoniac. Subsequent chemical discoveries came quickly, with Scheele [34] showing that it was present in the atmosphere, and Berthellot [35] demonstrating that it consisted of one part nitrogen to three parts hydrogen.

## 3. Ammonia and present-day changes in air pollution climate

**Figure 2.**
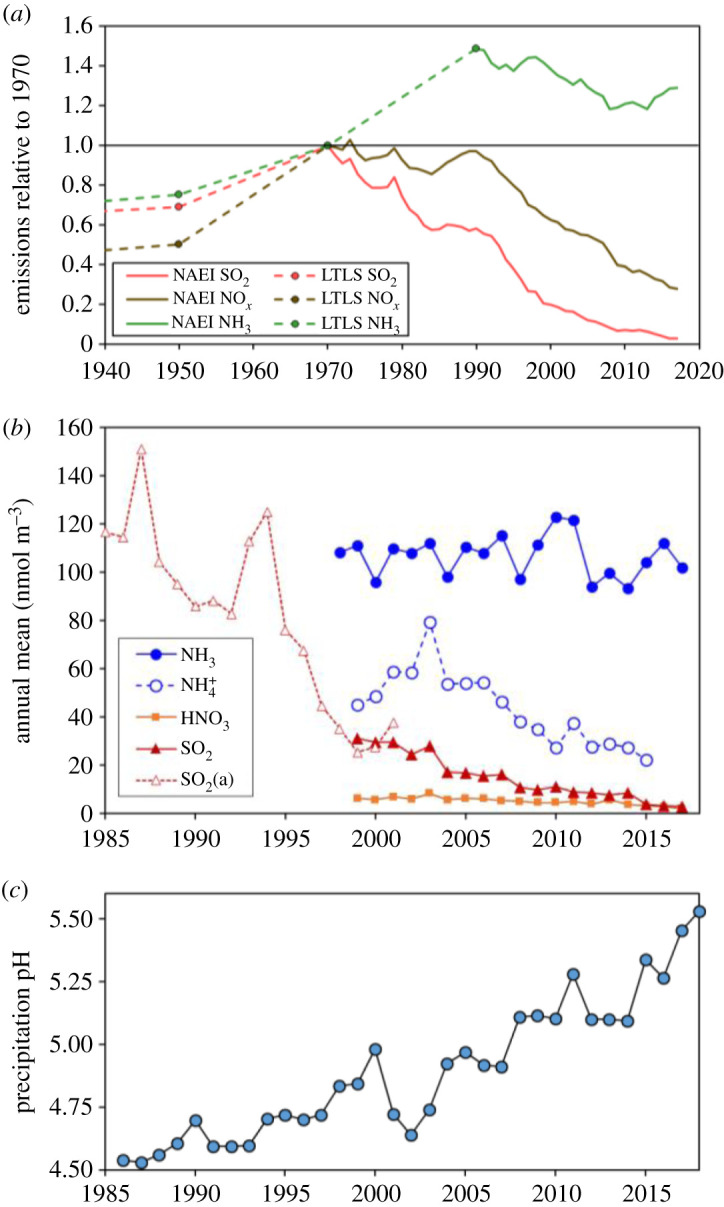
(*a*) Emissions of SO_2_, NO*_x_* and NH_3_ from the UK relative to 1970, comparing the Defra National Atmospheric Emissions Inventory (NAEI) including estimates from the Long-Term Large-Scale (LTLS) model for earlier years [36]. (*b*) Annual mean concentrations of gaseous NH_3_, SO_2_ and HNO_3_ and of aerosol NH_4_^+^ (for 12 sites), from the UK monitoring network (for further details and error analysis, see Tang *et al.* [37,38], compared with the earlier trend for five sites (SO_2_(a)), normalized to the UK mean for 1999–2001. (*c*) Volume-weighted mean pH of precipitation across the UK based on spatial interpolation of measured values.

The reminder of ammonia as alkaline air is highly relevant to the present, as emissions of SO_2_ and NO*_x_* have decreased greatly over the last 30 years, leaving European and North American atmospheres increasingly rich in NH_3_. This can be illustrated by the temporal evolution of emissions, gas and aerosol concentrations and rainfall acidity across the UK. While SO_2_ emissions have been almost entirely abated (97% reduction since 1970) and NO*_x_* emissions reduced by 70%, estimated NH_3_ emissions increased substantially up to 1990, decreased by 18% (1990–2013), and then increased 9% (2013–2017; figure 2*a*). National mean NH_3_ concentrations have not changed significantly since the National Ammonia Monitoring Network [37] was started in 1997 (though increasing in remote areas), while aerosol $\text{N}{{\text{H}}_{4}}^{+}$ concentrations have decreased significantly, consistent with declining SO_2_ and HNO_3_ (figure 2*b*). This has led to less formation of ammonium sulfate and ammonium nitrate, which will have also helped maintain gaseous NH_3_ levels [38,39].

**Figure 3.**
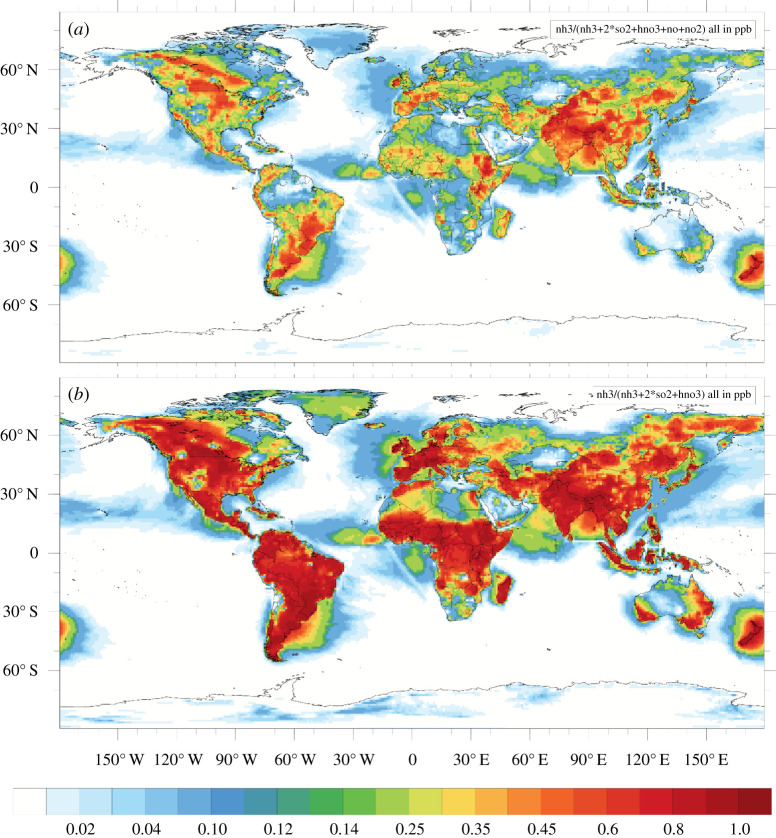
Global distribution of the gaseous alkaline fraction for 2010 as estimated by the EMEP-WRF global model [44], here calculated based on surface atmosphere mixing ratios (ppbv/ppbv) as NH_3_ / (NH_3_ + HNO_3_ + 2SO_2_ + NO*_x_*): (*a*) including NO*_x_*, (*b*) excluding NO*_x_*, since it is unclear to what extent NO*_x_* concentrations influence leaf surface acidity (see electronic supplementary material, §4).

As a consequence, acid rain is now a thing of the past for UK conditions. Since 1986, volume-weighted rain pH has increased from 4.62 to 5.48 (figure 2*c*), now being close to the value of 5.6 due to dissolution of atmospheric CO_2_. Together these changes demonstrate how alkaline air is becoming increasingly important across the UK countryside, in a pattern that is reflected across much of Europe and North America [40,41]. A corresponding trend is now occurring in China, following implementation of SO_2_ emission controls from 2012 [42], while in India, NO*_x_* emissions have been increasing even faster than NH_3_ emissions [43]. The gaseous alkaline fraction (expressed as NH_3_ divided by the sum of NH_3_, 2SO_2_, HNO_3_ and HCl) is now at 88% in the UK (electronic supplementary material, §4), while estimated global variation is shown in figure 3. In many areas of the world, the gaseous alkaline fraction is over 60% (including NO*_x_*) or 80% (excluding NO*_x_*).

The net result of these changes is that $\text{N}{{\text{H}}_{4}}^{+}$ is now making an increasing relative contribution to the composition of airborne particulate matter, relevant for effects on human health [45]. In parallel, the increasingly alkaline, NH_3_-rich atmosphere is having substantial consequences for the natural environment, as examined in detail below for lichens and other sensitive plants.

### (a) Response of lichens to atmospheric ammonia

While lichens are well known to be sensitive to SO_2_ concentrations, here we emphasize that NH_3_ is now the primary air pollution driver of lichen distributions in many areas of Europe. To understand the dynamics, we first consider a local-scale transect from Scotland [46] that shows how lichens can change in the vicinity of a poultry farm emitting NH_3_. Lichens on tree trunks of both Scots pine (*Pinus sylvestris*) and Sitka spruce (*Picea sitchensis*), and on branches of birch (*Betula pubescens*), which are all naturally acid-barked trees, were scored according to a standard methodology [47,48]. In this approach, lichen species are categorized as ‘acidophytes’ (e.g. *Usnea*, *Hypogymnia*, *Pseudevernia*, *Bryoria*), preferring naturally acidic bark, and ‘nitrophytes’ (e.g. *Xanthoria*, *Physcia*), favouring higher levels of nitrogen air pollution (electronic supplementary material, §3) [49]. Using this approach, frequency-based lichen indices for acidophytes (L_A_) and nitrophytes (L_N_) were calculated (see electronic supplementary material, §5), where the difference (L_AN_ = L_A_–L_N_) distinguishes bark dominated by acidophytes (+ value) or nitrophytes (− value).

**Figure 4.**
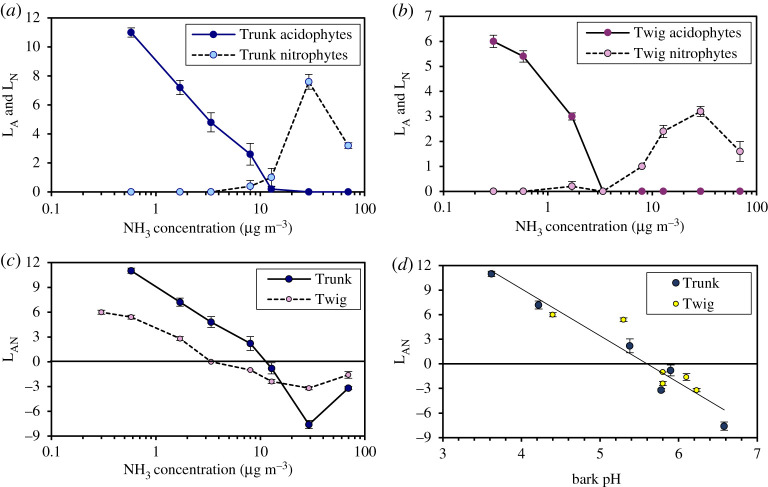
Response of epiphytic lichens on trunks and twigs of *Betula pubescens* to increasing NH_3_ concentrations in a farm transect in South Scotland. (*a*) trunks and (*b*) twigs, for a cover index of lichen acidophytes (L_A_) and nitrophytes (L_N_). (*c***)** Results for the joint index L_AN_ = L_A_ – L_N_. (*d*) Combined relationship between L_AN_ and bark pH for twigs and trunks: L_AN_ = − 5.73 (bark pH) + 32.1, with *R*^2^ = 0.91. Error bars are ±1 standard error for five replicate trees at each location. (Online version in colour.)

Findings from the local transect are summarized in figure 4, showing how acidophyte species were gradually eradicated between mean NH_3_ concentrations of 1 and 12 µg m^−3^, with acidophytes on twigs being more sensitive to NH_3_ than those on trunks. Acidophytes on both twigs and trunks were already significantly reduced at the third cleanest location (approximately 1.7 µg m^−3^, two-sample *t*-test, two-tail assuming unequal variance, trunks: *p* < 0.001; twigs: *p* < 0.01), where the first nitrophytes on twigs were also recorded. Highest nitrophyte occurrence was recorded at 30 µg m^−3^, with a significant reduction at 70 µg m^−3^ for both trunks (*p* < 0.001) and twigs (*p* = 0.01). Figure 4*d* shows that there was also a significant relationship between L_AN_ and measured bark pH. This effect can be largely explained by NH_3_ increasing bark pH nearer the farm (see electronic supplementary material, §5). It is notable that there is no significant difference in the relationship between L_AN_ and bark pH for twigs versus trunks (figure 4*d*). This indicates that the greater sensitivity of acidophyte lichens on twigs is consistent with the differences in bark chemistry between twigs and trunks. One of the advantages of the local study shown in figure 4 is that it covers a wide range of pollution levels from 0.3 to 70 µg m^−3^ demonstrating its wide relevance for different pollution conditions.

The lichen methodology was subsequently applied at 30 sites across the UK [50]. It must be recognized that different tree species also have naturally different bark pH, and therefore the analysis distinguished lichen communities on naturally acid-barked oak (*Quercus robor*, *Q. petraea*, recorded where available) from communities on other tree species. L_AN_ was generally not found to be correlated with SO_2_ concentrations (except for a weak relationship for oak trunks, *p* = 0.04, *n* = 11), with a lack of relationship with SO_2_ also found in a later survey [51].

**Figure 5.**
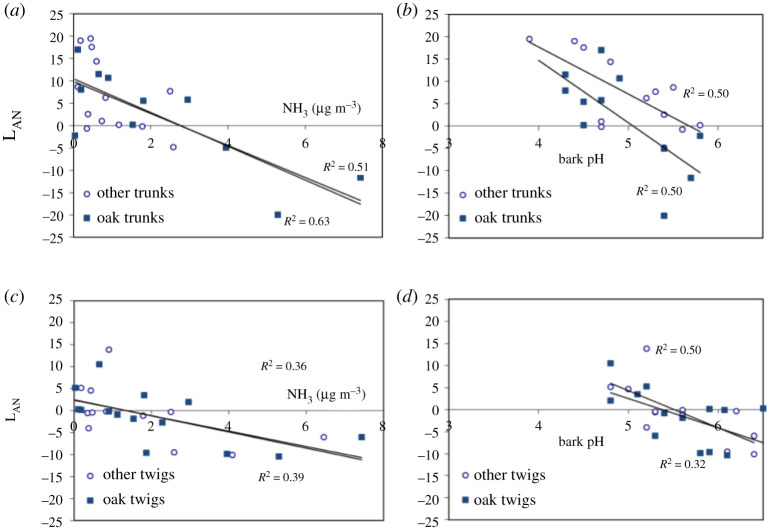
Relationship between epiphytic lichens on trunks and twigs of oak and other tree species to ambient NH_3_ concentrations (*a*,*c*) and bark pH (*b*,*d*) from 30 sites across the UK for trunks (*a*,*b*) and twigs (*c*,*d*). Results are shown for the joint index L_AN_ = L_A_ – L_N_, where L_A_ is the cover score for acidophyte lichens and L_N_ the score for nitrophytes. (Online version in colour.)

At the UK-scale, trunks and twigs both show reducing L_AN_ score with higher NH_3_ and with higher bark pH, demonstrating the broad relevance of these relationships (figure 5). Substantial scatter can be seen between NH_3_ concentration and L_AN_ score, which is expected based on natural variation in bark pH, even under clean conditions. In addition, variations with climate may have introduced some scatter. For example, precipitation was found to have a weakly significant effect for lichens on twigs (*p* < 0.05: *R*^2 ^= 0.15 (all data), *R*^2 ^= 0.35 (oak); electronic supplementary material, figure S4), but was not significant for lichens on trunks.

**Figure 6.**
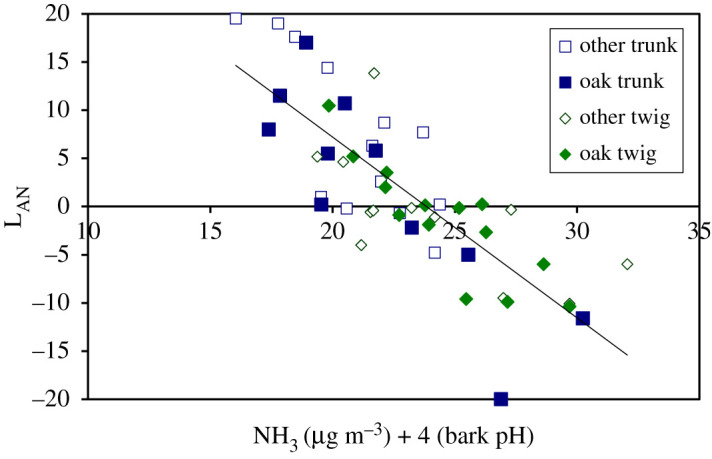
Relationship between the lichen index (L_AN_) and a combination of NH_3_ concentration and bark pH. L_AN_ = L_A_ – L_N_, where L_A_ and L_N_ are abundance indices for acidophyte and nitrophyte lichen species, respectively. Combining the trunk and twig data, the *R*^2^ values are 0.72 for lichens on oak (*n* = 25, *p* < 0.001), 0.56 for other trees (*n* = 25, *p* = 0.001) and 0.64 for all data (*n* = 50, *p* < 0.001), where L_AN_ = − 1.8771 [NH_3_ + 4 (bark pH)] + 44.752. (For further comparisons see electronic supplementary material, figure S4.) (Online version in colour.)

However, an even higher correlation was found for the UK by relating the L_AN_ scores to a combined index of NH_3_ (μg m^−3^) + 4 (bark pH) (figure 6). This points to NH_3_ as potentially having two effects. Firstly, NH_3_ has an alkaline effect, shown by its increasing bark pH (electronic supplementary material, figure S3A). Secondly, there appears to be an effect that is not explained by the changes in bark pH. If NH_3_ only had its effect by altering bark pH, then this would not explain why a combined index of NH_3_ and bark pH gives an improved relationship with L_AN_ than with bark pH alone. Other diversity and nitrogen indicators are illustrated in electronic supplementary material, §5 (electronic supplementary material, figure S3), with further statistical comparisons and the full UK dataset given in electronic supplementary material, figure S4 and table S6, respectively.

Comparable results have been recorded for The Netherlands [52], showing in particular a long-term decline of acidophyte lichen species from 1991 to 2016, consistent with differences in bark pH [49]. Since these datasets focus on the principles of lichen responses to acid and alkaline gases, similar relationships can be expected in other parts of the world. For example, extremely high levels of NH_3_ in the Indo-Gangetic Plain [14] can be expected to be adversely affecting acidophyte epiphytic lichens in the oak forests of the Himalayan foothills, which have significant economic importance, being traded to Arabic speaking countries to make valued-added products like perfumes [53]. While data on NH_3_ responses have not been available until now, first analysis as part of the GCRF South Asian Nitrogen Hub [54] shows major gradients in modelled NH_3_ and N wet deposition both N-S and W-E across sub-Himalayan forests and at levels that greatly exceed the known impact thresholds for lichens in temperate biomes.

### (b) Ecological effects of ammonia versus ammonium and nitrate

Differential sensitivity of vegetation according to the form of N air pollution is also indicated by the results of a long-term pollution manipulation experiment at Whim Bog in Southern Scotland [55,56]. In this globally unique experiment, the effects of gaseous NH_3_, assessed using free-air enrichment from a line-source of NH_3_, are compared with the effects of wet deposited $\text{N}{{\text{H}}_{4}}^{+}$ and $\text{N}{{\text{O}}_{3}}^{-}$, as delivered to replicated mesocosms (12.8 m^2^; four replicate plots for each N level/form combination) through a misting system (see electronic supplementary material, §6). Treatments at this site have been continuing for 18 years since 2002, allowing examination of the long-term effects of different N forms. Background deposition to the site is estimated at 8 kg N ha^−1^ yr^−1^, with treatments achieving total inputs of 16, 32 and 64 kg N ha^−1^ yr^−1^. Assessment of plant species composition is based on recording at three permanent quadrats for each experimental plot, with each quadrat divided into 16 sub-quadrats (see electronic supplementary material, §6).

**Figure 7.**
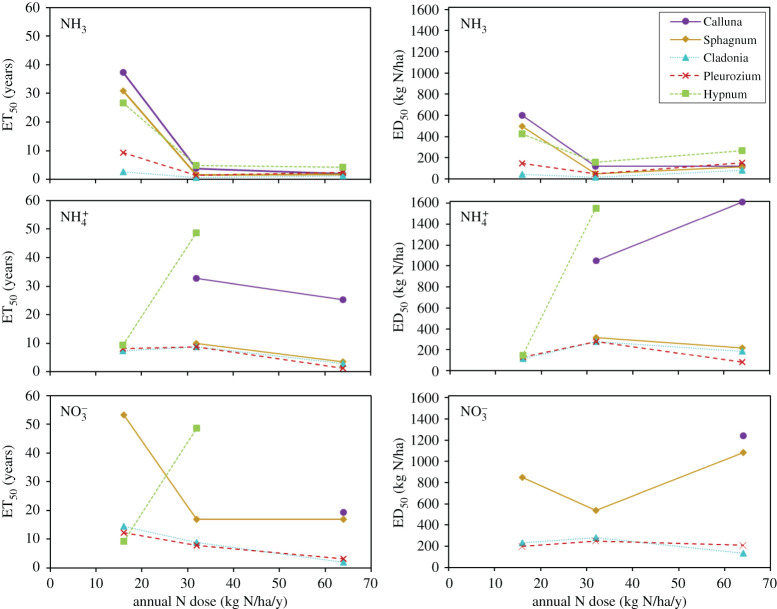
Response of bog vegetation to exposure of gaseous NH_3_ and wet deposited $\text{N}{{\text{H}}_{4}}^{+}$ and $\text{N}{{\text{O}}_{3}}^{-}$ expressed as the time taken to reduce cover of each plant species by 50% of initial values (Eradication Time 50%, ET_50_), and the Eradication Dose 50% (ED_50_) representing the total accumulated N dose that led to a halving of cover. The smallest values are most robust, with large values most uncertain, since these depend on extrapolation. (Online version in colour.)

The outcomes for changes in plant cover of the main species sensitive to N pollution are summarized in figure 7. This provides an analysis of ‘Eradication Time 50’ (ET_50_), which is defined here as the time taken to reduce species cover by 50% relative to cover at the start of the experiment. This is shown together with the ‘Eradication Dose 50’ (ED_50_), the cumulative N deposition over the period associated with a relative 50% reduction in cover of each species. The values of ED_50_ are calculated as the product of ET_50_ and the annual nitrogen inputs for 2002–2019 (see electronic supplementary material, figure S5 and table S6). Changes for other species included hare's-tail cotton grass (*Eriophorum vaginatum*), which benefited from NH_3_ relative to other, more-sensitive species [56].

Considering the species shown in figure 7, reindeer lichen (*Cladonia portentosa*) was found to be overall most sensitive, followed by red-stemmed feather-moss (*Pleurozium schreberi*) and red bog-moss (*Sphagnum capillifolium*). ET_50_ for NH_3_ at 32 kg N ha^−1^ yr^−1^ for these species were all estimated in the range of 0.6–1.6 years. By contrast, common heather (*Calluna vulgaris*) and heath plait-moss (*Hypnum jutlandicum*) were less sensitive to NH_3_, with ET_50_ values of 3.8–4.9 years, for the same N dose. Expressed as ED_50_, the most sensitive species had values of 19–51, while *Calluna* and *Hypnum* had values of 122 and 157 kg N ha^−1^ (electronic supplementary material, table S7).

Overall, it is expected that ET_50_ values are larger at lower annual N dose rates, as shown by the left side of figure 7. By contrast, it is expected that ED_50_ should be independent of annual N dose rate, so the extent to which this expectation is not met indicates that the ecological response is not directly proportional to accumulated N inputs.

As expected, ET_50_ values were larger at low N inputs and smaller at high N inputs. This is shown for impacts of NH_3_ (all species) and for impacts of wet deposited $\text{N}{{\text{H}}_{4}}^{+}$ and $\text{N}{{\text{O}}_{3}}^{-}$ for the most sensitive species (*Cladonia*, *Pleurozium*). By contrast, significant scatter is seen for *Calluna* and *Hypnum* in response to wet deposited N, reflective of longer and more uncertain ET_50_ estimates, with values greater than 17 years based on extrapolation of linear fits (electronic supplementary material, figure S5). Data for *Cladonia*, *Pleurozium* and to some extent *Sphagnum* give the best evidence for the utility of the ED_50_ indicator, as the plots for these species all show little difference between N dose rate, as expected compared with the ET_50_ indicator. By comparison, lower ED_50_ values for *Hypnum*, *Sphagnum* and *Calluna* for the NH_3_ treatments at 32 and 64 kg N ha^−1^ yr^−1^, as compared with the 16 kg N ha^−1^ yr^−1^ NH_3_ treatment, suggest that these species are more-than-proportionately vulnerable at the higher N rates. This could point to a toxic effect as a result of higher NH_3_ *concentrations* rather than dose.

**Figure 8.**
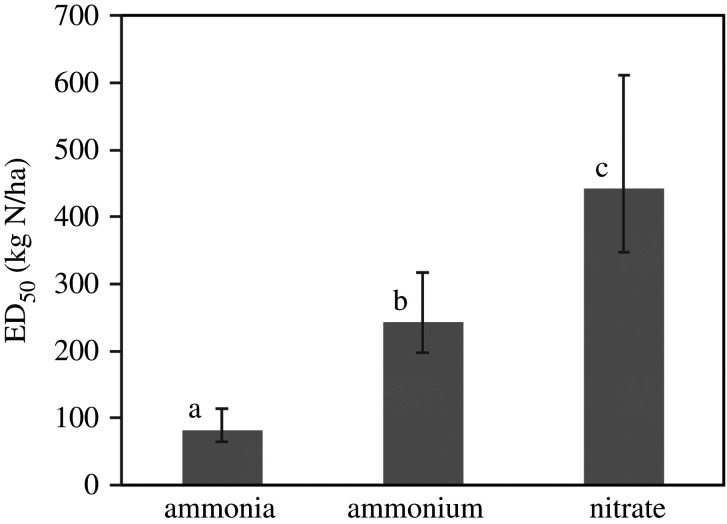
Differential sensitivity of bog vegetation to gaseous NH_3_ and wet deposited $\text{N}{{\text{H}}_{4}}^{+}$ and $\text{N}{{\text{O}}_{3}}^{-}$, expressed as the average Eradication Dose 50 (ED_50_). Mean and standard errors for five plant species at three N input levels (*n* = 15, figure 7 and electronic supplementary material, table S7). Different letters show statistical significance with *p* < 0.05 (two-tail); (*a,c*) are significantly different at *p* < 0.01, based on paired *t*-tests of the reciprocal values.

The most important observation from this dataset for the present analysis is that it further demonstrates the higher sensitivity to NH_3_ compared with wet deposited $\text{N}{{\text{H}}_{4}}^{+}$ and $\text{N}{{\text{O}}_{3}}^{-}$. This is most clearly seen by calculating statistics based on normalizing the data as 1/ED_50_, which also allows inclusion of all treatments, with the data then plotted as ED_50_ (figure 8). Overall, it can be seen that the reductions in cover of these five species occur three and five times faster for gaseous NH_3_ than for the same N dose of wet deposited $\text{N}{{\text{H}}_{4}}^{+}$ and $\text{N}{{\text{O}}_{3}}^{-}$, respectively. Comparable differences were also revealed by an independent assessment of physiological response for lichen transplants (electronic supplementary material, figure S6).

Although it is widely assumed that effects on plant nutrition are governed by total N inputs, our data thus show that reality is more complex, otherwise there would be no difference between the wet $\text{N}{{\text{H}}_{4}}^{+}$ and $\text{N}{{\text{O}}_{3}}^{-}$ treatments, as well as between the wet N and dry NH_3_ treatments. Other experimental studies have mainly been conducted using increased wet deposited N or fertilizer addition [57,58]. Had such studies included NH_3_ enhancement, they may therefore have found even larger adverse effects per unit N added. This is not to suggest that effects of wet deposited N are unimportant, but rather to emphasize the need to consider N form. For example, in agricultural areas subject to high NH_3_ concentrations, our results show that remaining bog habitats will be more than proportionately at risk (based on estimated N deposition). Conversely, wet deposited N may lead to larger effects on an area basis, since the largest areas of bogs are far from agricultural sources, where wet N deposition dominates total N inputs [56].

### (c) Recovery following reduction in ammonia concentrations

The preceding examples, showing higher sensitivity of plants to NH_3_ compared with $\text{N}{{\text{H}}_{4}}^{+}$ and $\text{N}{{\text{O}}_{3}}^{-}$, raise the question of whether there are also different rates of recovery following reductions in N pollution. It has been suggested that recovery in species composition and certain N pools may take several decades after a reduction in wet deposition of N [59]. This may be especially the case in slow-growing naturally acidic ecosystems, where N pools change slowly due to lack of removal by harvests and inhibition of denitrification [60,61].

There are no known experimental studies of the simultaneous reduction in NH_3_, $\text{N}{{\text{H}}_{4}}^{+}$ and $\text{N}{{\text{O}}_{3}}^{-}$ pollution for any ecosystem. However, field data from a site in Northern Ireland illustrate the potential for surprisingly rapid recovery following a reduction in gaseous NH_3_ concentrations. In this case study, a poultry farm 50 m west of Moninea Bog Special Area of Conservation (SAC) led to greatly increased concentrations of NH_3_ (10–40 µg m^−3^) compared with local and regional background values of 1.5 and 0.5 µg m^−3^. The lichens *Cladonia portentosa, C. uncialis* and *Sphagnum* species were largely eradicated on the bog within 400 m of the farm, with excessive growth of algae on trunks of *Betula pubescens* (50–100 m from the farm) replacing the natural acidophyte lichen flora [62].

**Figure 9.**
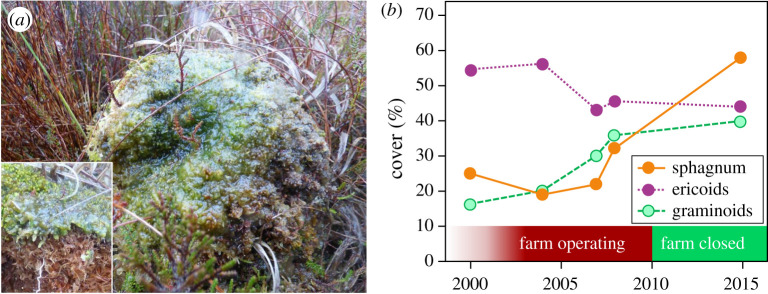
(*a*) Hummock of *Sphagnum* moss on Moninea Bog photographed in 2017, seven years after reduction in NH_3_ concentrations, showing a hummock still covered by algal slime characteristic of high NH_3_ levels (insert: partial cross-section). (*b*) Statutory monitoring showed an overall tendency for recovery in *Sphagnum* populations at Moninea Bog, but not yet of *Calluna* nor a return to previously lower levels of graminoids. These data confirm independent expert examination of the site by the authors in 2007 and 2017. (Online version in colour.)

As a consequence of legal requirements for the protection of Moninea Bog, the poultry farm ceased operation in 2010, allowing examination of ecosystem recovery. Observations in 2017 showed mean atmospheric NH_3_ concentrations of 1.5 µg m^−3^, with substantial recovery of *Cladonia portentosa* and *Sphagnum* spp. As all large clumps (*ca* 200–400 mm diameter) of *C. portentosa* had been eradicated, only uniformly small specimens (*ca* 50–70 mm) were found in 2017 in the zone where they had been previously eradicated. The extent of *Cladonia* and *Sphagnum* growth indicated that recovery must have started within 2–4 years of the reduction in NH_3_ concentrations. Conversely, it was not obvious whether the condition (i.e. overall health) of *Calluna* had improved, while residual algae levels on *Betula* less than 100 m from the farm indicated only partial recovery. An illustration of a potential ‘alternative stable state’ [59] was also found (figure 9*a*). This showed continued colonization of a *Sphagnum* hummock by algae, 7 years after NH_3_ concentrations reduced. It suggests ongoing competition, where a coating of gelatinous algal slime restricts gas exchange and growth of the *Sphagnum* until the latter can manage to grow over the algae. Monitoring across Moninea Bog by the Northern Ireland Environment Agency confirmed the recovery of *Sphagnum* after 2010, with no evidence of any recovery in *Calluna* (figure 9*b*). The latter effect may be age-dependent, where recovery of old *Calluna* plants (weakened or dead) is limited, while ultimately conditions may favour recolonization by young *Calluna* plants.

## 4. Discussion

### (a) Mechanisms of ammonia impacts on vegetation

The examples presented highlight the increased sensitivity of lichens and bog vegetation to gaseous NH_3_ compared with wet deposited $\text{N}{{\text{H}}_{4}}^{+}$ and $\text{N}{{\text{O}}_{3}}^{-}$, which appears to be at least partly related to the alkaline effect of NH_3_. In this way, higher correlations were found between L_AN_ and NH_3_ in the UK-scale lichen survey than with total N deposition. In fact, the highest single-factor correlation was found with the ratio of N to S deposition (electronic supplementary material, figure S4). This indicator is also closely correlated with NH_3_ concentrations (*R*^2 ^= 0.87), and, like NH_3_, also provides an indication of acid-base balance.

Both the national and local-scale lichen surveys showed that bark pH is positively correlated with NH_3_ concentration, while L_AN_ score is negatively correlated with bark pH. Hence, one of the ways in which NH_3_ appears to affect epiphytic lichens is by increasing substrate pH. That this is one of the driving variables is also shown by the differences between tree species, with nitrophytes found to be more prevalent on trees with naturally higher bark pH. Natural differences in bark pH can similarly explain differences in lichen communities between twigs and trunks (figures 4*d* and 6). This raises the question of whether the NH_3_ effect on lichens is *entirely* mediated by its effect on surface pH.

Examination of the UK-scale data suggests a more complex interaction. If NH_3_ has its effect solely through changing bark pH, then differences in pH should fully explain the variation in L_AN_ with NH_3_. This would therefore not explain why a combined indicator of NH_3_ + 4 (bark pH) gives a better relationship with L_AN_ than with bark pH alone (figure 6; electronic supplementary material, figure S4). It suggests that NH_3_ may be affecting lichens by both a pH effect and another effect, such as that related to nutrient N (as more usually considered to drive N effects on ecosystems [57]).

**Figure 10.**
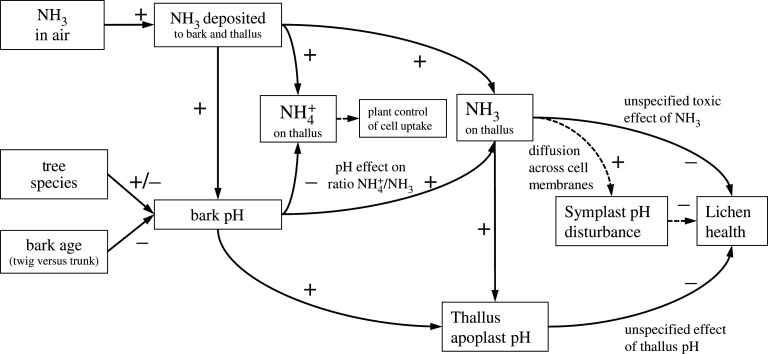
Possible mechanisms by which atmospheric NH_3_ pollution affects epiphytic lichens, including both positive (+) and negative (−) effects. Solid lines indicate observed relationships or those directly implied by physico-chemistry. Dashed lined indicate hypothesized relationships. The toxic and pH effects apply especially to acidophyte lichens, but may also apply to nitrophyte lichens at high levels of NH_3_ exposure (figure 4).

Possible relationships are summarized in figure 10: NH_3_ deposited on bark increases $\text{N}{{\text{H}}_{4}}^{+}$ on the lichen thallus, uptake of which will be under control of cell membranes. Such altered nutrient supply may affect competition between species. In parallel, the deposition adds NH_3_ to the bark/thallus surface, which increases bark pH because of the alkaline nature of NH_3_. The bark pH is also affected by tree species and bark age (twig versus trunk). With NH_3_ increasing bark pH, the chemical equilibrium favours NH_3_ rather than $\text{N}{{\text{H}}_{4}}^{+}$, which further increases NH_3_ levels on the thallus. Two subsequent effects may then be expected. Firstly, that changed bark pH affects apoplast pH of the lichen thallus (cf. electronic supplementary material, figure S7), which could affect lichen health (e.g. by affecting the buffering systems of lichen acids characteristic of different species). Secondly, NH_3_ may have a direct toxic effect, including that mediated by the passive diffusion of NH_3_ across cell membranes, leading to disturbance of symplastic pH. That plant sensitivity to NH_3_ and other forms of N deposition is partly due to different abilities of species to manage cell pH homeostasis has been argued by Pearson & Soares [63], who elsewhere demonstrated a positive correlation between leaf buffering capacity index and nitrate reductase (NR) activity across 18 plant species [64]. This would offer another reason why acidophyte lichens, adapted to $\text{N}{{\text{H}}_{4}}^{+}$ nutrition (with low NR activity expected), would be more vulnerable to atmospheric NH_3_.

Effects of NH_3_ on lichen pH are also seen at Whim Bog. Electronic supplementary material, figure S8 shows that NH_3_ increased the pH of transplanted *Cladonia portentosa* thalli, confirming the thallus pH effect (figure 10), with responses seen within one month of transplantation. By contrast, the surface pH of live *Sphagnum capillifolium* growing in-situ remained unaffected, which may reflect a greater water-holding and buffering capacity of *Sphagnum*.

The importance of such pH effects may also explain the rapid recovery of *Cladonia portentosa* and *Sphagnum* spp. following reduction in NH_3_ levels at Moninea Bog. Even though the peat might still contain high N levels, reduced alkalinity from less NH_3_ would be expected to allow rather rapid re-adjustment of surfaces, allowing colonization of acidophyte species.

While uncertainties remain over the exact mechanisms, the higher sensitivity to NH_3_ compared with wet deposited $\text{N}{{\text{H}}_{4}}^{+}$ observed at Whim Bog tends to support this picture. Based on the values of ED_50_ (with NH_3_ being three times more damaging than NH_4_^+^), this suggests that ¾ of the NH_3_ effect on peatland vegetation could be related to pH effects, while ¼ of the NH_3_ effect is attributable to the common effect of increased nutrient N supply. One of the implications of our findings is therefore to pay more attention to the ‘critical level’ for NH_3_ concentrations for which the UNECE has adopted a value of 1 µg m^−3^ for lichens, bryophytes and associated habitats [65,66].

The extent to which such relationships can be generalized between species, habitats and world regions remains an important question for further work. Each species responds individually according to its nitrogen and pH preferences, sensitivity to NH_3_ toxicity and ability to compete with other species for light and other resources. For example, investigations on *Cladonia portentosa* from Whim Bog showed that different N forms affect different metabolic pathways [67,68], which may have varying importance between species. It is also possible to identify useful functional groups, as illustrated by the nitrophyte/acidophyte lichen groupings. *Calluna vulgaris* offers another illustration as this is found to be more sensitive to NH_3_ at Whim Bog than Cross-Leaved Heath (*Erica tetralix*) [56]. If it could be shown (according to [64]) that this reflects a lower apoplastic buffering capacity of *Calluna* than *Erica*, then this would encourage further use of buffering capacity as a predictive indicator. In the same way, species/group differences in characteristic lichen acids may also point towards predictive capability with global relevance, which may be tested by the GCRF South Asian Nitrogen Hub.

### (b) The future of alkaline air and nitrogen policy

The higher sensitivity of vegetation to gaseous NH_3_ compared with wet deposited NH_4_^+^ and $\text{N}{{\text{O}}_{3}}^{-}$ has direct implications for the success of past SO_2_ and NO*_x_* emission reductions in protecting ecosystems. While the acid rain problem has now been addressed in the UK and most of Europe, the modest reductions in NH_3_ emissions mean that alkaline air is emerging as a new ecological challenge. The data presented here focus on naturally acidophyte species, which appear to be especially vulnerable to alkaline air. It remains to be tested whether naturally basic habitats, such as chalk grasslands, would be less vulnerable to ammonia.

Already there are indications that NH_3_ concentrations are actually increasing in some parts of Europe rather than decreasing. While this is partly related to reduced SO_2_ and NO*_x_* concentrations leading to increased NH_3_ lifetimes, as reflected in NH_3_ monitoring for remote areas [38], there is also concern about climate change impacts on NH_3_ concentrations. As most NH_3_ globally results from volatilization processes, climate warming will increase NH_3_ emissions [69,70]. Strategies to address alkaline air therefore need to include measures that both reduce NH_3_ emissions directly [71] and minimize climate change drivers. In addition to control of CO_2_ and CH_4_ emissions, decreasing losses of all N compounds (including N_2_O, NO and N_2_ to air, and $\text{N}{{\text{O}}_{3}}^{-}$ losses to water) becomes critical to increasing economy-wide nitrogen use efficiency, with multiple benefits for climate, air quality, water quality, biodiversity and stratospheric ozone protection [54,72]. Such a perspective could help transform current efforts to meet the EU National Emission Ceilings commitments for 2030, as well as many other policy goals.

This takes us closer to developing the big idea whereby ammonia becomes a key focus in an emerging international strategy to manage the global nitrogen cycle. This is why the historical perspective of §2 is so important, in raising awareness about ammonia. One of the lessons of history is that ammonia has always been of significant societal importance. From its role as part of the alchemists' objective to prepare Gold and the Elixir of Life, to its economically high value as a luxury product of international trade, ammonia continues today to be important in sustaining humanity through nitrogen fertilizers and biological nitrogen fixation. If society is to learn to manage nitrogen better, then these stories can help by raising wider awareness.

Ultimately, it may be the economic value of nitrogen that counts most. It has been estimated that global N losses to the environment amount to around 200 million tonnes [73,74]. This means that at a nominal market price of US$1 per kg N, a goal to ‘halve nitrogen waste’ from all sources by 2030 would offer a circular economy opportunity worth US$100 billion per year, amounting to an annual saving of approximately 12 kg N per person (cf. §2a). These issues have recently been recognized in the first Resolution on Sustainable Nitrogen Management adopted at the UN Environment Assembly (UNEP/EA.4/Res.14), with the ambition to halve nitrogen waste adopted in the Colombo Declaration [75]. The follow-up to these activities is bringing ammonia and air pollution together as part of the global nitrogen challenge, by working to establish an Interconvention Nitrogen Coordination Mechanism (INCOM), with targeted science support through the International Nitrogen Management System (INMS) [54,72]. Together these activities can be expected to emphasize how ammonia and the wider nitrogen cycle must be at the heart of the solutions needed for both environment and economy in working towards the UN Sustainable Development Goals.

## Supplementary Material

Alkaline Air: changing perspectives on nitrogen and air pollution in an ammonia-rich world. Supplementary Material.

## Data accessibility

Data associated with this paper are included in the electronic supplementary material.
