# Supplementary material for: Alkaline air: changing perspectives on nitrogen and air pollution in an ammonia-rich world
Source: Philos Trans A Math Phys Eng Sci. 2020 Sep 28;378(2183):20190315. doi: 10.1098/rsta.2019.0315 (PMC7536028; doi:10.1098/rsta.2019.0315)
Supplement: Alkaline Air: changing perspectives on nitrogen and air pollution in an ammonia-rich world. Supplementary Material. [file rsta20190315supp1.pdf]

Alkaline Air: changing perspectives on nitrogen and air pollution in an ammonia-rich world.

Supplementary Material.

Philosophical Transactions of the Royal Society A.

DOI: 10.1098/rsta.2019.0315

Mark A. Sutton[1], Netty van Dijk[1], Peter E. Levy[1], Matthew R. Jones[1], Ian D. Leith[1], Lucy J. Sheppard[1], Sarah Leeson[1], Y. Sim Tang[1], Amy Stephens[1], Christine F. Braban[1], Ulrike Dragosits[1], Clare M. Howard[1], Massimo Vieno[1], David Fowler[1], Paul Corbett[2], Mohd Irfan Naikoo[3], Silvana Munzi[4,5], Christopher J. Ellis[6], Sudipto Chatterjee[7] Claudia E. Steadman[8], Andrea Móring[8] and Patricia A. Wolseley[9]

1, UK Centre for Ecology & Hydrology, Edinburgh Research Station, Bush Estate, Penicuik, UK.
2, Northern Ireland Environment Agency, Belfast, UK.
3, Department of Botany, Aligarh Muslim University (AMU), Aligarh, India.
4, Centro Interuniversitário de História das Ciências e da Tecnologia, Faculdade de Ciências, Lisbon, Portugal.
5, Centre for Ecology, Evolution and Environmental Changes, Faculdade de Ciências, Lisbon, Portugal.
6, Royal Botanic Garden Edinburgh (RBGE), Edinburgh, UK.
7, Department of Natural Resources, TERI School of Advanced Studies (TERISAS), New Delhi, India.
8, School of Geosciences, University of Edinburgh, UK.
9, Natural History Museum, Cromwell Road, London, UK.

Contents

Supplementary Section 1: Comparison of nitrogen with other commodities 2
Supplementary Section 2: Long-term changes in nitrogen prices 4
Supplementary Section 3: Ammonia in Greek alchemical literature 8
Supplementary Section 4: Air quality changes and Alkaline Air 10
Supplementary Section 5: Lichen responses to atmospheric ammonia 12
Supplementary Section 6: Effects of controlled ammonia exposure on peatland vegetation .. 18
Supplementary References 27
Appendix A: A Greek list of alchemical names for aqueous ammonia 30

Supplementary Section 1: Comparison of nitrogen with other commodities

Central Asia (7th century)

Records from a year's trade at a customs station from c. 620 have been preserved because the paper was reused as grave shoes in Turfan (Tang, 1992). Based on the data provided by Skaff (1998, p 91) concerning the 'scales tax', it is possible to calculate the price of sal ammoniac and compare this with the price of spice and silk. The calculation is based the assumption that a 0.9% scales tax for silver applied equally for all commodities. The calculations for *nao sha* were checked using a transaction of Shemipantuo to Kang Yanyuan for a combined sale of spice and *nao sha*, which agreed within 10%. Table S1 assumes that brass and copper were taxed at 1/10 of the price for silver, with sugar and medicine taxed as for spices. The main uncertainties in Table 1 (main text) result from: i) gaps in the annual register, ii) whether the 0.9% tax rate (Skaff, 1998, p 93) applied equally to all commodities and, iii) the extent to which the slave price (Skaff, 1998, p 89; Hansen, 2005, p 295) was representative.

Just five luxury goods represented over 90% of the trade in weighed goods. While spice was the most important commodity, *nao sha* (sal ammoniac) was the second most important, with 444 kg traded in just six transactions. The price of *nao sha* was similar to silk at around 6 pure silver (*nuqra*) dirhams per kg. Combining these data with the recorded slave price, it can be calculated that 22 kg of *nao sha* (equivalent to 6 kg of nitrogen) would have bought a high quality slave.

Egypt (11th – 12th centuries)

Among the extensive records from the Cairo Geniza, Goitein (1999, p 228) summarizes 12 transactions for the export of sal ammoniac, mainly bound for Sicily and Tunisia (Supplementary Table S1). Goitein notes stable rates of exchange, where 1 gold dinar = 13 1/3 *nuqra* (pure silver) dirhams or 36 low-quality silver dirhams (the usual coin in Cairo), with a *mann* of 2 Fustat pounds (*ratl*) representing ~450 g (pp 368-392).

The spice referred to in the Cairo data is pepper, with silk mainly sold by weight (pp 221-222). As in Turfan, the price of a slave is for a female slave (where the standard rate recorded in the Cairo Geniza was 20 dinars; mean is 18.2±2.62 dinars, 95% confidence limits, n=19). Only one record gives the price of a male slave, at 19.5 dinar (Goitein, 1999, pp 133-139).

Sal ammoniac rates are averaged as kg/dirham to normalize and reflect purchasing practice (0.132±0.038 kg *nūshādir* / *nuqra* dirham; equivalent to 0.0346±0.0010 kg N/*nuqra* dirham; 95% confidence limits, n=12). Nitrogen rate for slaves in Table 1 combines the relative confidence limits for nitrogen and slaves as square root of the sum of the squares.

The availability and conservative exchange of the *nuqra* dirham in both contexts makes it possible to compare the Central Asian and Egyptian situations.

Table S1: Trade in *nao sha* (sal ammoniac) and other weighed commodities recorded in one year (c. 620) from a weighing station near Turfan. Calculations are based on records summarized by Skaff (1998, pp 81-93).

Commodity	No. trans-actions	Total weight of all transactions		Total 'scales tax' collected		Commodity unit price [b]	Price of a female slave [b,c]
	(#)	(kg)	(% share)	(*nuqra* dirham)	(% share)	(*nuqra* dirham/kg)	(kg commodity /slave)
Spice	9	1433	66	71	46	6	22
nao sha	6	444	20	22.5	14	6	22
silver	6	10.1	0.5	21	13	228	0.5
silk thread	5	120	6	17.5	11	17	7
gold	6	2.4	0.1	13	8	626	0.2
other [a]	5	160	7	12	8	8.5	14
Total	37	2170	100	157	100		

Notes: **a**, Single transactions for medicine, muscovado sugar, turmeric, brass and copper; **b**, Assuming the 'scales tax' of 0.9% for silver applied to all weighed commodities; **c**, Based on a slave from Samarkand sold in Turfan in 639.

Table S2: Records of *nūshādir* (sal ammoniac) sale from the Cairo Geniza. Calculations based on records summarized by Goitein (1999, pp 228-229).

Record number	Date	Destination of sale	Price paid (Gold dinar)	*nūshādir* Weight (mann)	*nūshādir* Rate (mann/ dinar)	*nūshādir* Rate (kg /dinar)	*nūshādir* Rate (kg/*nuqra* dirham)	*nūshādir* Price (*nuqra* dirham/kg)	Comment
1	1000	Tunisia	1.00	2.88	2.88	2.59	0.19	5.15	"no demand"
2	1020	Qayrawān Tunisia	1.00	2.50	2.50	2.25	0.17	5.93	
3	1030	Sicily	1.00	2.00	2.00	1.80	0.14	7.41	
4	1035	Al-Mahdiyya Tunisia	57.30[a]	109.25[a]	1.90	1.71	0.13	7.78	
5	1050	Old Cairo	27.00	50.00	1.85	1.67	0.13	8.00	
6	1052	Qayrawān Tunisia	1.13	1.00	0.89	0.80	0.06	16.67	"prices were low"
7	1055	Qayrawān Tunisia	1.00	1.33	1.33	1.20	0.09	11.11	"an excellent price"
8	1060	Mazara Sicily	1.00	1.38	1.38	1.24	0.09	10.77	
9	1063	Al-Mahdiyya Tunisia	1.00	2.00	2.00	1.80	0.14	7.41	
10	1070	Al-Mahdiyya Tunisia	1.00	2.00	2.00	1.80	0.14	7.41	
11	1075	Al-Mahdiyya Tunisia	0.50	2.00	4.00	3.60	0.27	3.70	prices were "quiet"
12	1140	Alexandria	1.00	0.75	0.75	0.68	0.05	19.75	"prices like fire"
**Mean**	**1054**				**1.96**	**1.76**	**0.132**	**7.57**[b]	
Standard Error					0.256	0.230	0.0173		

Notes: **a**, Based on the mean of three transactions; **b**, Derived as the reciprocal from the mean rate (kg/*nuqra* dirham).

Supplementary Section 2:
Long-term changes in nitrogen prices

Comparison of N prices through centuries is complicated by different rates of exchange between multiple currencies. To simplify the comparison, Figure S1 shows N prices normalized to the nominal prices of gold and silver. Table S3 shows the data on which these estimates are based. Figure S1 indicates that, relative to the price of silver, nitrogen compounds (mainly ammonium, ammonia and urea) are currently about 2-3 orders of magnitude cheaper than the earliest records. Relative to the price of gold, present-day nitrogen compounds are 3-4 orders of magnitude cheaper than the early records. The highest relative price for nitrogen noted here concerns purchase (c. 1500) of sal ammoniac among a wide range of alchemical ingredients used at the Scottish royal court (Read, 1938).

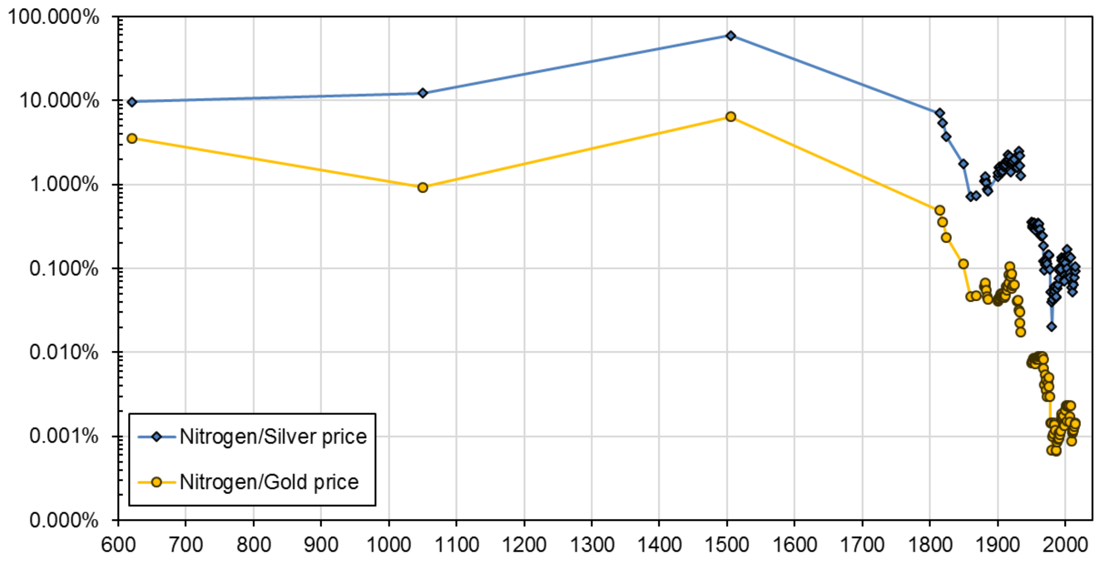

Figure S1: Changing price of nitrogen over the centuries indicated by the ratio to contemporary silver and gold prices (For data see Tables S3 and S4).

Table S3: Comparison of nitrogen price over the centuries with gold and silver prices – based on nominal price (For basis see Table S4).

Year	Silver price	Gold price	Nitrogen price	Price units	Nitrogen/Silver price (%)	Nitrogen/Gold price (%)
620	228	626	22.9	silver dirhams (*nuqra*) / kg	9.7%	3.58%
1050	235	3,133	28.9	"	12.3%	0.92%
1505	24.7	229.3	14.7	Pound Scots/kg	59.6%	6.42%
1814	10.2	147.0	0.718	Pound Sterling/kg	7.064%	0.488%
1819	9.0	138.2	0.489	"	5.444%	0.354%
1824	8.1	128.0	0.301	"	3.705%	0.235%
1849	8.8	138.2	0.155	"	1.771%	0.112%
1860	8.9	137.0	0.0637	"	0.712%	0.047%
1868	8.7	135.9	0.0637	"	0.732%	0.047%
1880	36,970	664,230	408	US dollar /tonne	1.104%	0.061%
1881	36,330	664,230	409	"	1.126%	0.062%

Year	Silver price	Gold price	Nitrogen price	Price units	Nitrogen/Silver price (%)	Nitrogen/Gold price (%)
1882	36,650	664,230	450	"	1.228%	0.068%
1883	35,690	664,230	370	"	1.037%	0.056%
1884	35,690	664,230	310	"	0.869%	0.047%
1885	34,080	664,230	285	"	0.836%	0.043%
1900	20,000	609,000	250	"	1.25%	0.041%
1901	19,000	610,000	260	"	1.37%	0.043%
1902	17,000	609,000	270	"	1.59%	0.044%
1903	17,000	609,000	280	"	1.65%	0.046%
1904	19,000	608,000	290	"	1.53%	0.048%
1905	20,000	607,000	300	"	1.50%	0.049%
1906	22,000	608,000	300	"	1.364%	0.049%
1907	21,000	609,000	300	"	1.429%	0.049%
1908	17,000	609,000	290	"	1.706%	0.048%
1909	17,000	609,000	275	"	1.618%	0.045%
1910	17,000	608,000	275	"	1.618%	0.045%
1911	17,000	608,000	290	"	1.706%	0.048%
1913	19,600	608,000	370	"	1.89%	0.061%
1914	18,000	610,000	338	"	1.88%	0.055%
1915	16,400	616,000	370	"	2.26%	0.060%
1916	21,500	623,000	423	"	1.97%	0.068%
1917	27,000	629,000	536	"	1.98%	0.085%
1918	31,500	635,000	673	"	2.14%	0.106%
1919	36,000	641,000	510	"	1.42%	0.080%
1920	32,800	660,000	573	"	1.75%	0.087%
1921	20,300	662,000	381	"	1.88%	0.058%
1922	21,900	667,000	414	"	1.89%	0.062%
1924	21,500	665,000	431	"	2.00%	0.065%
1929	17,000	663,000	271	"	1.59%	0.041%
1930	12,200	662,000	278	"	2.28%	0.042%
1931	9,320	723,000	232	"	2.49%	0.032%
1932	9,000	665,000	200	"	2.23%	0.030%
1933	11,300	847,000	191	"	1.69%	0.023%
1934	15,400	1,120,000	195	"	1.27%	0.017%
1950	23,800	1,120,000	84.8	"	0.356%	0.0076%
1951	28,600	1,120,000	87.5	"	0.306%	0.0078%
1952	27,300	1,110,000	87.5	"	0.320%	0.0079%
1953	27,300	1,120,000	95.3	"	0.349%	0.0085%
1954	27,300	1,130,000	95.3	"	0.349%	0.0084%
1955	28,600	1,130,000	93.6	"	0.327%	0.0083%
1956	29,300	1,130,000	82.6	"	0.282%	0.0073%
1957	29,300	1,120,000	92.5	"	0.316%	0.0083%
1958	28,600	1,130,000	92.5	"	0.323%	0.0082%
1959	29,300	1,130,000	94.7	"	0.323%	0.0084%
1960	29,300	1,130,000	101	"	0.346%	0.0090%
1961	29,600	1,130,000	101	"	0.342%	0.0090%
1962	35,000	1,130,000	101	"	0.290%	0.0090%

Year	Silver price	Gold price	Nitrogen price	Price units	Nitrogen/Silver price (%)	Nitrogen/Gold price (%)
1963	41,200	1,130,000	101	"	0.246%	0.0090%
1964	41,500	1,130,000	101	"	0.244%	0.0090%
1965	41,500	1,130,000	101	"	0.244%	0.0090%
1966	41,500	1,130,000	101	"	0.244%	0.0090%
1967	49,800	1,130,000	92.5	"	0.186%	0.0082%
1968	68,800	1,290,000	83.7	"	0.122%	0.0065%
1969	57,550	1,330,000	55.0	"	0.096%	0.0041%
1970	56,910	1,170,000	62.7	"	0.110%	0.0054%
1971	49,830	1,330,000	61.6	"	0.124%	0.0046%
1972	54,010	1,880,000	66.1	"	0.122%	0.0035%
1973	82,310	3,140,000	93.6	"	0.114%	0.0030%
1974	151,400	5,140,000	220	"	0.145%	0.0043%
1975	142,100	5,190,000	204	"	0.144%	0.0039%
1976	139,900	4,030,000	204	"	0.146%	0.0051%
1977	148,500	4,770,000	143	"	0.097%	0.0030%
1978	173,600	6,220,000	90.3	"	0.052%	0.0015%
1979	356,600	9,890,000	143	"	0.040%	0.0014%
1980	663,300	19,700,000	135	"	0.020%	0.0007%
1981	338,200	14,800,000	146	"	0.043%	0.0010%
1982	255,600	12,100,000	129	"	0.050%	0.0011%
1983	367,800	13,600,000	196	"	0.053%	0.0014%
1984	261,700	11,600,000	159	"	0.061%	0.0014%
1985	197,400	10,200,000	119	"	0.061%	0.0012%
1986	175,900	11,800,000	79.2	"	0.045%	0.0007%
1987	225,400	15,400,000	105	"	0.046%	0.0007%
1988	209,900	14,100,000	120	"	0.057%	0.0009%
1989	176,800	12,300,000	114	"	0.065%	0.0009%
1990	155,000	12,400,000	117	"	0.075%	0.0009%
1991	130,000	11,700,000	129	"	0.099%	0.0011%
1992	127,000	11,100,000	117	"	0.092%	0.0011%
1993	138,000	11,600,000	134	"	0.097%	0.0012%
1994	170,000	12,400,000	232	"	0.137%	0.0019%
1995	166,000	12,400,000	210	"	0.127%	0.0017%
1996	167,000	12,500,000	209	"	0.125%	0.0017%
1997	157,000	10,700,000	191	"	0.122%	0.0018%
1998	178,000	9,490,000	134	"	0.075%	0.0014%
1999	169,000	9,000,000	120	"	0.071%	0.0013%
2000	161,000	9,010,000	186	"	0.115%	0.0021%
2001	141,000	8,750,000	202	"	0.143%	0.0023%
2002	149,000	10,000,000	151	"	0.101%	0.0015%
2003	158,000	11,700,000	270	"	0.171%	0.0023%
2004	215,000	13,200,000	301	"	0.140%	0.0023%
2005	236,000	14,300,000	334	"	0.142%	0.0023%
2006	373,000	19,500,000	333	"	0.089%	0.0017%
2007	431,000	22,500,000	338	"	0.078%	0.0015%
2008	482,000	28,100,000	650	"	0.135%	0.0023%

Year	Silver price	Gold price	Nitrogen price	Price units	Nitrogen/Silver price (%)	Nitrogen/Gold price (%)
2009	472,000	31,300,000	277	"	0.059%	0.0009%
2010	644,000	39,500,000	437	"	0.068%	0.0011%
2011	1,130,000	50,600,000	585	"	0.052%	0.0012%
2012	1,000,000	53,800,000	638	"	0.064%	0.0012%
2013	767,000	45,500,000	596	"	0.078%	0.0013%
2014	623,000	40,800,000	584	"	0.094%	0.0014%
2015	505,000	37,400,000	530	"	0.105%	0.0014%

Table S4: Basis for the comparison on nitrogen prices to silver and gold prices (Figure S1 and Table S3).

Year	Substance	Source of data
620	sal ammoniac, silver, gold	Turfan; Customs register (Table S1)
1000-1140	sal ammoniac, silver, gold	Cairo; Trade records from the Cairo Geniza (Table S2)
1505	sal ammoniac, silver, gold	Scotland; Records of the court alchemist to King James IV of Scotland (Holmyard, 1957; Read, 1938).
19th C	silver, gold	International; The Denver Gold Group Inc. (2017) Historical Gold and Silver Prices (in USD). http://www.denvergold.org/precious-metal-prices-charts/historical-gold-prices/ https://www.denvergold.org/precious-metal-prices-charts/historical-silver-prices/ (US to UK exchange rates from: https://fred.stlouisfed.org/series/USUKFXUKA)
1814	sal ammoniac	Scotland; Report of Sir John Sinclair, cited by Clow and Clow (1952, p 421).
1819, 1824	sal ammoniac	India; Anon (1819, p 415; 1825, p 111)
1849	ammonium carbonate	England; Anon (1853, p 263).
1860-1868	sal ammoniac, ammonium sulphate	USA; Commissioner of Agriculture (1870, p 292).
1880-1885	ammonium sulphate, sodium nitrate	USA; Kunsman (1940, p 10).
20th C	silver, gold	USA; US Geological Service. https://minerals.usgs.gov/minerals/pubs/historical-statistics/
1913-1922	anhydrous ammonia, ammonium sulphate, sodium nitrate	USA; US Department of Labor, Bureau of Labor Statistics (1923, p 169).
1924-1933	anhydrous ammonia, ammonium sulphate, sodium nitrate	USA; US Department of Commerce, Statistical Abstract of the United States (1935, p 296), Table No. 332.- Wholesale prices of leading commodities.
1950-present	anhydrous ammonia	USA; US Geological Service. https://minerals.usgs.gov/minerals/pubs/historical-statistics/

Supplementary Section 3:
Ammonia in Greek alchemical literature

Over the last century, it has been most common to assume that ammonia and ammonium were not known in the Greek alchemical literature characteristic of c. 100 BC to 700 AD. Instead, it has been suggested that they were first known to Arabic and Persian alchemists after around 800 AD (e.g., Ruska, 1923; Needham *et al.*, 1980, p 435). Stapleton (1953, p 40) was unusual in supporting earlier Greek alchemical knowledge of sal ammoniac, noting reference to it in a treatise (in later Arabic translation) attributed to the Greek alchemist Zosimus, who flourished c. 300 AD. By contrast, Hallum (2008) considered that this reference represented a later interpolation by an Arab scribe/editor. While Hallum may be correct as such, this does not distract from the important point the supposed Arab scribe was making: "It is *nushādir*, I believe, truly, truly" ('Tome of the Art', line 48, trans. Hallum, 2008, p 362). At minimum, the implication is that the Arab scribe considered the Greeks to have been familiar with ammonium salt. The text, as translated by M. Turāb 'Ali (version corrected by H.E. Stapleton, 1948) reads as follows, with Theosebia addressing Zosimus:

> She said: "Inform me about Nushadur (Sal-Ammoniac)".
>
> He replied: It is a bird that flies without a wing. It is called "The Eagle" on account of its flying into the air (when heated)…
>
> She said: "Inform me about the Generous Stone which the Sages have mentioned".
>
> He replied: It is a Noble Stone. I will not call it by its name, but if you (so) desire, I will let you know its signs, its benefits and its names, without naming it.
>
> She said: "Do so".
>
> He replied: It is a Stone and not a Stone. It has a Spirit, a Soul and a Body. If it is liquefied, it becomes liquefied. If it is coagulated, it becomes coagulated. It is of white colour, spacious, swift, light and sultry [or 'briny']: the tongue cannot describe it. In my opinion it is Nushādur. Verily, verily, I have described it as being with the rich, the poor and all people – in markets, on roads and in dung heaps. None of the Sages can do without it in any of their operations…
>
> You should not be deceived by the numerous descriptions of the Sages: because in the "Stone of the People" there will be (found) Four Natures, in the analysis by fire, and Three by appearance, every one of them surrounding its companion. So ponder over this and pay no attention to the unnecessary things which the Sages have inserted in their books in order to conceal it from the (common) people. And I have named it to you by its true name (TS Stapleton 104, pp 4-7, History of Science Museum, Oxford).

Other arguments for Greek alchemical knowledge of ammonia and ammonium include:

a) Several other Arab/Persian authors attribute earlier use to the Greeks, including: al-Jāhiz (Wiedemann, 1907, pp 76-77) and al-Rāzī ('Book of Evidences', see Stapleton and Azo, 1910). The same is seen in an anonymous Greek text that relates organic alchemical distillation to the four 'elements', Water, Earth, Fire, Air. Although Colinet (2000) concluded that this work was originally written in Arabic, synonyms added by the Greek editor attribute the procedures as being of prior Greek origin (see Appendix A).

b) Several Greek alchemical texts describe processing of organic matter, where 'cover names' (*Decknamen*) demonstrate how organic substances may also be present in other books. For example in a text 'On the same divine water', attributed to Zosimus (Mertens, 1995, pp 30-33), the first fraction of distillation (ammonium carbonate) is referred to as 'rain water' (i.e., macrocosmic allusion), with subsequent distillation fractions referred to as 'radish oil' and 'castor oil'. The philosophical perspective of this work is illustrated by the reference to the awful smell, none of which should escape, because it is "the first-principle of the art (*huparchei hē technē*)", applying a characteristic Neoplatonic term.

c) Several processes seen in Islamic alchemy are recognizable in preceding Greek alchemy. Although the earlier texts are deliberately more obscure, it is often possible to identify contrasting descriptions of the same process. This is illustrated by the process of al-Rāzī (Section 2.2): the tincture of burnt oil (Fire) is extracted along with the acid by suspending the coagulated oil in ammonia / ammonium carbonate solution (Water), then repeating 5-6 times, thereby 'marrying' the Fire and Water. The same process can be found in an Arabic translation of Zosimus (*Muṣḥaf aṣ-ṣuwar*, Abt, 2011, pp 486-492), including reference to the suspended matter, repeated washing, marriage and colours.

The alchemists' multiple description of the same process with different 'cover names' appears to be a characteristic approach of gaming in early alchemical literature, where texts represent literary puzzles to be solved. Contrary to Hallum (2009), the *Muṣḥaf aṣ-ṣuwar* is here noted to meet all the criteria of an authentic (if edited) translation of Zosimus (e.g., all appropriate substances and authorities named or not named). However, in case this should be a point of contention, it is worth noting that the same alchemical marrying of Water and Fire can be seen in the *Phusika kai Mustika* traditionally attributed to Democritus (Martelli, 2013, pp S86-95). Here the procedure is repeated in ten short operations using apparently different ingredients (concerning which Zosimus dedicated a 10-chapter commentary; Abt, 2016). For example, an operation of Democritus to "make cinnabar white", uses "oil, or vinegar, or honey, or brine, or alum", where these five substances appear to be cover names for five additions of the ammonia solution, which extract the redness, so whitening the coagulated oil (the Cinnabar). The subsequent recipe even mentions "castor oil or radish oil".

It should be emphasized that this is not a new interpretation, but was the traditional reading of the alchemists themselves. In the margin of a famous alchemical manuscript (Venezia, Biblioteca Nazionale Marciana, Gr. Z. 299 (=584), see f. 68 v.) formerly owned by Cardinal Basilios Bessarion (likely brought with him from Constantinople, c. 1438), Bessarion (?) commented of the Cinnabar-whitening above: "Alum is aether and ☽ [Mercury (*hudrarguros*, literally water-silver)] and copper-without-shadow", succinctly demonstrating his understanding of these synonyms. It suggests we should "sharpen our mind" (Synesius, Commentary on the Book of Democritus, trans. Martelli, 2013, p S129) to the idea that ammonium, *nūshādir*, aether and alum may all be one thing, their meaning summarized lightly in the overall synthema ✳, a form applicable to all.

While such recipes of the earlier Greek alchemical literature have been interpreted over the past century as technical procedures using the chemicals named, the perspective outlined here thus indicates that they are better understood as presenting a few covert processes in multiple ways, as playful puzzles to be decoded. This suggests that first appearances of key features in alchemical literature (e.g., new substances, processes and philosophical interpretations) may not actually be new at all. Rather, it seems more likely that such innovations mainly reflect *increasing openness* in the description of existing alchemical activity and of its meaning as understood by the alchemists. As a result, we may expect to find ammonia processes in many other alchemical writings, including the exuberant periphrasis of Stephanus of Alexandria (Papathanassiou, 2017) and the Greek alchemical poems, such as that attributed to Heliodorus (Goldschmidt, 1923). A further conclusion, that both ammonia and alchemy are similarly recognizable in the extant philosophical fragments of Empedocles and Heraclitus and in several romances, such as the Aethiopica of Heliodorus and the tale of Cenerentola, is likely to be contentious and must be a matter for future debate. This perspective highlights the extreme difficulty and uncertainty in tracing early knowledge of ammonia.

Supplementary Section 4: Air quality changes and Alkaline Air

Whereas 'alkaline air' was the original name given to gaseous ammonia by Priestley, the main text (Section 1) also defines alkaline air from an environmental perspective as "air where alkaline gases (primarily NH_3, but in principle also including volatile amines) dominate over those that are acidic in nature (primarily SO_2, NO_x, HCl etc)."

Based on this concept, we define an additional term the 'gaseous alkaline fraction' in air based on the relative composition of alkaline and acidic components. Values larger than 0.5 can be considered as representing 'alkaline air' from this environmental perspective.

The total of gaseous alkaline components ($\Sigma_{alkaline}$) and acidic components (Σ_{acid}) is summed to give a combined total ($\Sigma_{alk+acid}$), calculated as follows (expressed in nmol m^{-3} or ppb):

$$\Sigma_{alk+acid} = NH_3 + 2\ SO_2 + HNO_3 + HCl + NO_x \qquad [E1]$$

For the present calculation, $\Sigma_{alkaline}$ is simply taken as the gaseous NH_3 concentration in air (nmol m^{-3} or ppb). As SO_2 is oxidized in the atmosphere (and when deposited on surfaces) to H_2O_4 it is considered as dibasic. The value of $\Sigma_{alk+acid}$ may be calculated including or excluding NO_x, since it is rather insoluble in water, so that its effect (especially when deposited on plant surfaces) may be much less in practice than indicated by NO_x concentration. Other components typically existing at lower concentrations, such as amines, nitrous acid and organic acids could also be included in a more comprehensive approach where data are available. The approach could similarly be extended to consider the contribution of non-neutral particulate matter, including calcareous particles (i.e., as total alkaline fraction), as well as the contribution of increasing CO_2 concentrations.

Based on values of $\Sigma_{alk+acid}$, the gaseous alkaline fraction (θ_{alk}) is calculated simply as:

$$\Theta_{alkaline} = \Sigma_{alkaline} / \Sigma_{alk+acid} \qquad [E2]$$

Application of this approach to the UK (see Figure S2), indicates that $\Theta_{alkaline}$ has increased from 30% in 1985 to 88% in 2017 (when excluding NO_2). Including NO_2 in the calculation of $\Theta_{alkaline}$ gives values ranging from 18% in 1997 to 37% in 2017. Based on the assumption that NO_2 concentration mainly represents potential rather than effective acidity (especially when considering immediate effects on leaf surfaces), the mean UK atmosphere can be considered as having represented alkaline air ($\Theta_{alkaline}$>50%) since 1997. An alternative way to express such differences would be to calculate Θ values based on the relevant dry deposition fluxes to ecosystems.

Values of $\Theta_{alkaline}$ for the globe (Figure 3) are based on modelled gaseous concentrations for the surface atmosphere (derived from the model first layer 0 - 45 m) using the EMEP-WRF global model (Vieno *et al.*, 2016) – an implementation of the EMEP MSC-W model (Simpson *et al.*, 2012) using meteorology from the WRF model (Skamarock *et al.*, 2019). Global emissions for 2010 are taken from the ECLIPSEv6a emissions inventory (IIASA – GAINS model). HCl is not simulated in this model and is excluded from the estimation. The effect of including or excluding NO_x concentrations is shown in Figure 3A and 3B.

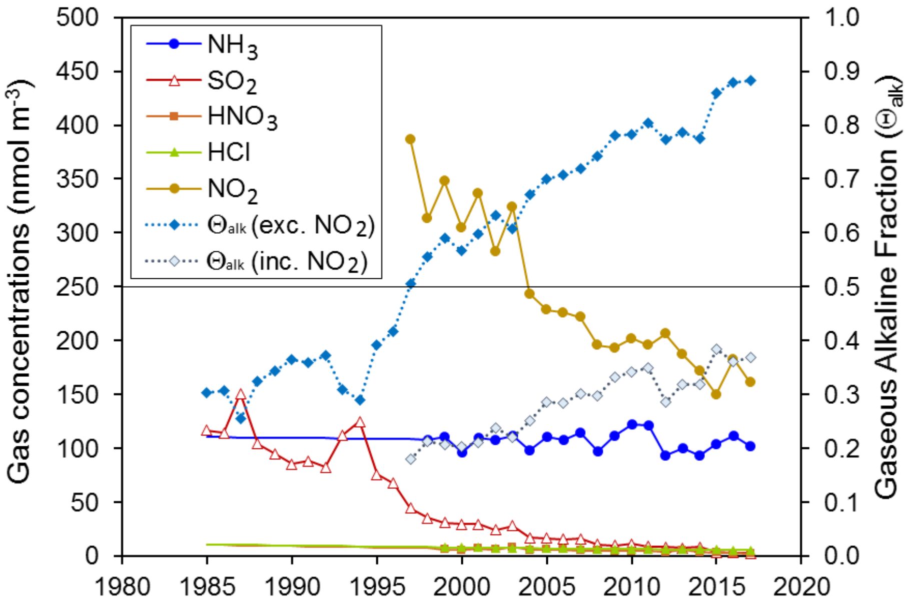

Figure S2: Temporal changes in mean air pollutant concentrations for the UK compared with the estimated gaseous alkaline fraction ($\Theta_{alkaline}$), shown both excluding and including NO_2. Values of NH_3, HNO_3 and HCl prior to 1997-1998 are based on extrapolation using linear least squares regression of the available data. Values of SO_2 1985-1999 are for 5 sites, normalized to the values for 12 sites (1999-2017) for the period 1999-2001 (Figure 2). Values for NO_2 are the means for rural background sites as reported by Defra (2019).

Supplementary Section 5: Lichen responses to atmospheric ammonia

Methodology for Lichen Recording at the Farm Transect

A transect was established in 2002 in a mixed woodland adjacent to a poultry farm near Earlston, southern Scotland, in order to assess the effects of atmospheric NH_3 on epiphytic lichen communities. Passive diffusion tubes were set up to monitor NH_3 concentrations on a transect through the woodland at 16, 46, 76, 126, 200 and 276 m from the poultry buildings in a northerly direction (Sutton *et al.*, 2004, pp. 75-86). For other nitrogen indicators at this transect, see Pitcairn *et al.* (2002, 2006). At each location established for NH_3 monitoring, 5 trees of *Pinus sylvestris* or *Picea sitchensis* with girths >40 cm were selected for recording lichens on trunks using a ladder quadrat of 5, 10 x 10 cm square quadrats placed on 4 cardinal aspects (North, South, East, West) between 1-1.5 m height on the trunk and lichens recorded in each quadrat. Frequency of epiphytic lichens occurring on twigs of *Betula pubescens* was recorded using presence on 5 sampled twigs in the vicinity of NH_3 sampling sites, as well as at a nearby nature reserve Gordon Moss (5.8 km) as a background site. Bark pH was recorded for each aspect on the trunks using a flat tip electrode, averaged for each tree and this was adapted to record bark pH on branches (Wolseley *et al.* 2006). Means were calculated for each sampling location. Crustose, fruticose and foliose lichens were recorded in all quadrats. Using indicator species identified by van Herk (2000), nitrophytes were distinguished from acidophytes. Based on the indices for acidophytes (L_A) and nitrophytes (L_N), the combined index L_{AN} was calculated as $L_{AN} = L_A – L_N$.

Methodology for Lichen Recording at the UK scale

The UK wide survey of epiphytic lichens was conducted in 2004 in the vicinity of National Ammonia Monitoring Network Sites (Tang *et al.*, 2019a). This combined intensive recording of epiphytic lichens at a few sites with a simplified recording of macrolichens, with fieldwork undertaken by agency staff at 32 sites across the UK (Wolseley *et al.*, 2005). Recording methods were the same as those used in the Earlston farm transect, combining bark pH with frequency of macrolichen species on trunks and twigs of trees adjacent to the NH_3 monitoring sites (Wolseley *et al.* 2009). Further research investigating the effects of NH_3, atmospheric pollutants and climatic factors on epiphytic lichens was undertaken during a PhD project (Lewis, 2012) leading to the preparation of a Nitrogen Air Quality Index (NAQI) using a Lichen Indicator score (Wolseley *et al.* 2013).

Supplementary Results from the Farm Transect

As shown in Figure S2, bark pH of both trunks and twigs was found to be positively correlated with NH_3 concentrations (A). Other relevant indicators measured at this site recorded trunk lichen diversity according to a German protocol (VDI, 1995) and European Protocol (LDV) (Asta *et al.*, 2000) (B), mean score according to Ellenberg nitrogen values of species present (Wirth, 1992) (C), and measured foliar nitrogen concentration of two key species, *Hypogymnia physodes* and *Xanthoria parietina* (D). While neither the VDI nor the LDV lichen diversity indices were found to correlate uniformly with the full range of NH_3 concentrations, positive correlations were seen for both the Ellenberg nitrogen index and foliar N concentration.

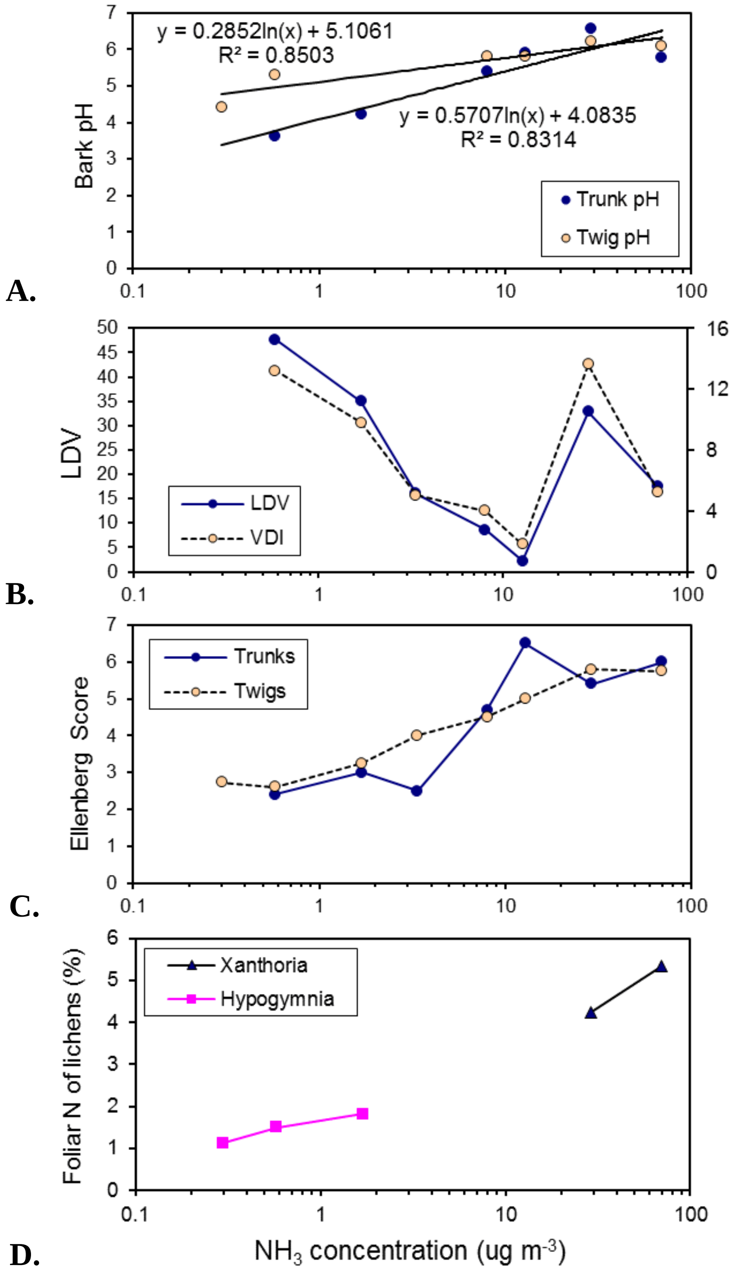

Figure S3: Relationships between measured NH3 concentrations and a range of chemical and biological indicators. A, bark pH; B, Lichen diversity according to German (VDI) and European (LDV) methods; C, Mean Ellenberg Nitrogen value of species present; D. Foliar Nitrogen concentration of collected lichen samples.

Supplementary Results from the UK-wide lichen survey

The full dataset for the UK-wide survey is shown in Table S5, from which the main relationships were derived based on the L_{AN} indices for trunks and twigs. Of the 32 sites, it was possible to obtain lichen survey data at 30 sites, allowing calculation of L_{AN} values at 24 sites for trunks (11 of which were for oak) and 26 sites for twigs (14 of which were for oak). Where it was possible to combine twig and trunk results, this provided 50 samples overall and 25 samples for oak. A summary of the main relationships between L_{AN} values possible drivers is given in Figure S4 expressed as correlation coefficients (R^2).

Consistently higher correlations were found when considering only the data for lichens growing on oak bark, compared with the full dataset, which can be explained by natural

difference in bark pH (reflective of different bark chemistry) between tree species. Higher correlations were found between L_{AN} and NH_3 concentrations than with N deposition.

Weak or insignificant relationships were found for L_{AN} with mean SO_2 concentrations and annual precipitation, with higher correlations with bark pH (significant in all tests shown in Figure S4). The highest correlation for a single factor relationship was found for the ratio of N deposition to S deposition, reflecting a strong negative correlation with N deposition and a weak positive correlation (only significant for twigs) of S deposition to L_{AN}. Although harder to understand than NH_3 concentrations, this suggests itself as another indicator of atmospheric acid-base relations. Indeed, the N deposition to S deposition ratio was found to be positively and closely correlated with NH_3 concentrations (R^2=0.87, n=32).

Figure S4 also shows the correlations with two combined two-factor indicators, where the correlations between L_{AN} and NH_3 + 4 (bark pH) are all highly significant. The correlations for LAN and N_{dep}/S_{dep} + 2.8 (bark pH) are slightly higher than for NH_3 + 4(bark pH). Again this suggests that the N_{dep}/S_{dep} indicator has additional utility as a complement to the simpler indicator of NH_3 concentrations. The multipliers 4 and 2.8 used in the combined pollution–bark indices are the values that maximize the overall correlation with L_{AN}.

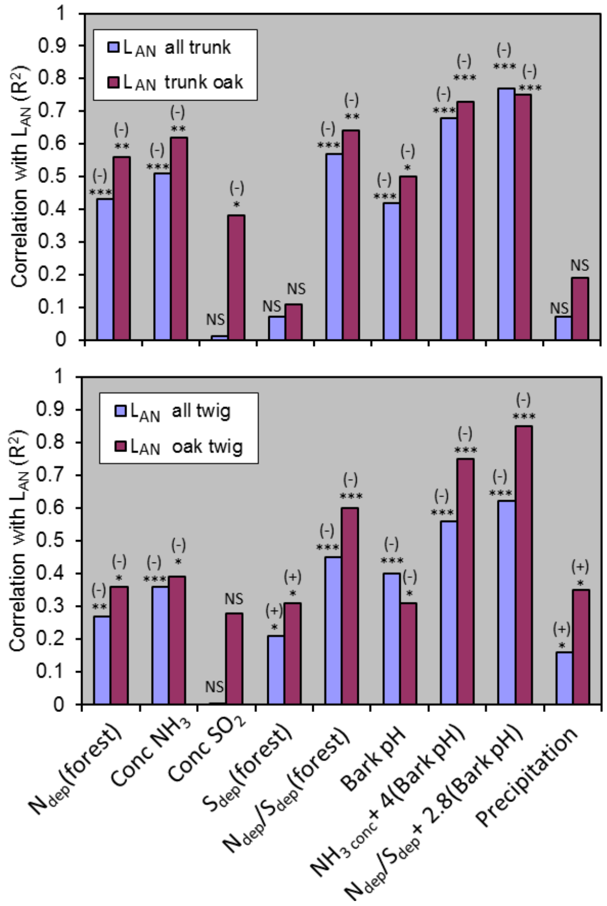

Figure S4: Summary of relationships between the lichen N air pollution index (L_{AN}) data collected at the UK-scale and a range of possible driving variables. Asterisks indicate significance: ***, P<0.001; **, P<0.01; *, P<0.05; NS, not significant. Bracketed + or – indicates a positive or negative relationship. Exclusion of one outlier for SO_2 concentrations (Edinburgh centre) would give 0.23 (all trunks, NS) and 0.05 (all twigs, NS).

Table S5: Summary of data for the local-scale lichen survey at Earlston (sites 1-6) and Gordon (site 7). Values of L_A, L_N and L_{AN} are updated compared with Sutton *et al.* (2009). Errors are ± standard errors for 5 replicate trees.

			Foliar N (% dry matter)		Trunks								Twigs					
Site	Distance from farm edge (m)	NH_3 conc. ($\mu g\ m^{-3}$)	*Xanthoria parietina*	*Hypogymnia physodes*	Tree species	Trunk pH	Lichen diversity VDI method	Lichen diversity LDV method	Ellenberg N index	Trunk L_N	Trunk L_A	Trunk L_{AN}	Tree species	Ellenberg N index	Twig pH	Twig L_N	Twig L_A	Twig L_{AN}
1	10	69.6	5.33	-	*Picea*	5.78	5.2	17.4	6	3.2 ±0.2	0.0 ±0.0	-3.2 ±0.2	*Betula*	5.4	6.1	1.6 ±0.4	0.0 ±0.0	1.6 ±0.4
2	16	29.13	4.24	-	*Pinus*	6.58	13.6	32.8	5.4	7.6 ±0.5	0.0 ±0.0	-7.6 ±0.5	*Betula*	5.2	6.23	3.2 ±0.2	0.0 ±0.0	3.2 ±0.2
3	46	12.8	-	-	*Pinus*	5.9	1.8	2.2	6.5	1.0 ±0.6	0.2 ±0.2	-0.8 ±0.7	*Betula*	5	5.8	2.4 ±0.2	0.0 ±0.0	2.4 ±0.2
4	76	8	-	-	*Pinus*	5.38	4	8.6	4.7	0.4 ±0.4	2.6± 0.8	2.2 ±0.9	*Betula*	3.7	5.8	1.0 ±0.0	0.0 ±0.0	1.0 ±0.0
5	126	3.37	-	-	*Pinus*		5	16	2.5	0.0 ±0.0	4.8 ±0.7	4.8 ±0.7	*Betula*	4.2	-	0.0 ±0.0	0.0 ±0.0	0.0 ±0.0
5a	200	1.7	-	1.81	*Pinus*	4.22	9.8	35	3	0.0 ±0.0	7.2 ±0.5	7.2 ±0.5	*Betula*	2.4	-	0.2 ±0.2	3.0 ±0.1	2.8 ±0.2
6	276	0.58	-	1.5	*Pinus*	3.62	13.2	47.6	2.4	0.0 ±0.0	11.0 ±0.3	11.0 ±0.3	*Betula*	2.1	5.3	0.0 ±0.0	5.4 ±0.2	5.4 ±0.2
7	5,800	0.3	-	1.13	-	-	-	-		-	-	-	*Betula*	2.1	4.4	0.0 ±0.0	6.0 ±0.2	6.0 ±0.2

Table S6: Summary of data for the UK-wide lichen survey. For further information, see Leith *et al.* (2005).

Site no.	Site name	National Grid Reference	Site operator	Habitat type	NH_3 conc. ($\mu g\ m^{-3}$)[1]	SO_2 conc. ($\mu g\ m^{-3}$)[2]	N deposition to woodland (kg N/ha/y)[2]	S deposition to woodland (kg S/ha/y)[2]	Ratio N/S deposition to woodland[2]	Precipitation[3]	Tree species	Trunk pH	Mean Trunk L_A	Standard dev. of Trunk L_A	Mean Trunk L_N	Standard dev. of Trunk L_N	Mean Trunk L_{AN}	Twig pH	Mean Twig L_A	Standard dev. of Twig L_A	Mean Twig L_N	Mean Trunk L_{AN}	Mean Twig L_{AN}
1	Cwmystwyth	SN772743	Ray Woods: CCW	Acid grassland	2.96	1.5	50.1	15.52	3.23	1540	*Quercus robor/petraea*	4.7	5.8	2.8	0	0	5.8	4.8	2.00	1.07	0	0.00	2.0
2	Plynlimon	SN822841	Ray Woods: CCW	Upland Heath	0.6	1.5	24.1	18.4	1.31	1939	*Sitka spr*	4.8*	14.4	2.3	0	0	14.4	4.8*	-	-	-	-	-
3	Dyffryn Mymbyr	SH695572	Alex Turner: CCW	Wet Acid Heath	1.18	1.3	47.5	23.36	2.03	3816	*Acer pseudoplatanus*	5.8	0.2	0.45	0	0	0.2	5.8*	-	-	-	-	-
4	Stackpole	SR983950	Bob Haycock: CCW	W8 lowland woodland	1.81	2	28.4	12	2.37	1018	*Quercus robor/petraea*	4.5	5.5	4.65	0.00	0.00	5.5	5.1	5.50	4.65	2.00	0.82	3.5
5	Eskdalemuir	NT235032	Chris Miles: SNH	Upland moorland	0.38	1.1	20.8	9.28	2.24	1556	*Populus nigra*	5.4	2.6	2.3	0.00	0.00	2.6	5.2	1.00	0.70	5.00	2.35	-4.0
6	Halladale	NC902488	Sandy Payne: SNH	Improved grassland	0.83	1.2	17.3	12.48	1.39	1181	*Betula pubescens*	5.2	6.3	9.30	0.00	0.00	6.3	5.6	0.50	0.84	0.67	0.52	-0.17
7	Strathvaich Dam	NH347750	J. Clayton, E. Turner: SEPA	Upland moorland	0.18	0.6	13.4	20.16	0.66	1882	*Betula pubescens*	4.4	19	0.00	0.00	0.00	19	4.8	6.17	2.93	1.00	0.63	5.17
8	Glensaugh	NO664799	J. Clayton, E. Turner: SEPA	Upland moorland	0.35	1.1	19.0	9.12	2.08	972	*Sorbus aucuparia*	5.6	0	0.00	0.70	0.58	-0.7	5.3	0.20	0.45	0.80	0.84	-0.6
9	Knockan	NC187088	Jan Breckenridge: SNH	Upland moorland	0.12	0.8	8.1	11.04	0.73	1464	*Sorbus aucuparia*	5.5	8.7	6.60	0.00	0.00	8.7	5.3	0	0	0	0	-
10	Edinburgh Centre	NT254738	I. Leith, N. van Dijk: CEH	Parkland	2.5	16.6	43.5	24.8	1.76	911	*Fraxinus excelsior*	5.3	7.7	5.69	0.00	0.00	7.7	6.2	0	0	0.33	0.52	-0.33
11	Ariundle	NM835642	Brian Eardley: SNH	Atlantic oak woodland	0.04	1.4	11.3	22.24	0.51	2608	*Quercus robor/petraea*	5.8	0.4	0.55	2.60	3.78	-2.2	5.2	5.60	5.94	0.40	0.55	5.2
12	Glen Nant	NN014278	Janie Steele: FC	Oak woodland	0.06	1.6	17.7	23.84	0.74	2588	*Quercus robor/petraea*	-	-	-	-	-	-	-	-	-	-	-	-
13	Wood of Cree	NX385715	Tommy Docherty: SNH	Atlantic oak woodland	0.12	1.5	16.2	12.8	1.27	1571	*Quercus robor/petraea*	4.7	21.7	7.51	4.70	5.51	17	6.5	0.22	0.67	0	0	0.22
14	Bush	NT245635	I. Leith, N. van Dijk: CEH	Parkland	0.9	3.7	27.1	12.8	2.12	1080	*Quercus robor/petraea*	4.9*	10.7	8.96	0.00	0.00	10.7	6.07	0.83	0.75	1.00	0.63	-0.17
15	Ladybower	SK163905	Richard Pollitt: EN	Blanket mire + wet & dry heath	0.73	5	51.5	30.56	1.69	1614	*Betula pubescens*	4.7	1	1.73	0.00	0.00	1	5.5	0	0	0	0	-
16	Moorhouse	NY751334	Bart Donato: EN	Blanket mire	0.44	2.2	39.6	20.64	1.92	1437	*Larix europaea*	3.9	19.5	1.00	0.00	0.00	19.5	5	4.63	2.56	0	0	4.63

Site no.	Site name	National Grid Reference	Site operator	Habitat type	NH_3 conc. ($\mu g\ m^{-3}$)[1]	SO_2 conc. ($\mu g\ m^{-3}$)[2]	N deposition to woodland (kg N/ha/y)[3]	S deposition to woodland (kg S/ha/y)[3]	Ratio N/S deposition to woodland[3]	Precipitation	Tree species	Trunk pH	Mean Trunk L_A	Standard dev. of Trunk L_A	Mean Trunk L_N	Standard dev. of Trunk L_N	Mean Trunk L_{AN}	Twig pH	Mean Twig L_A	Standard dev. of Twig L_A	Mean Twig L_N	Mean Trunk L_{AN}	Mean Twig L_{AN}
17	Fenn's Moss	SJ478368	Joan Daniels: EN	Raised bog	1.87	2.2	36.82	8.16	4.51	825	*Quercus robor/petraea*	5.4	0	0.00	0.00	0.00	-	5.9	0.60	0.55	10.20	3.56	-9.6
18	Stanford	TL858948	Peter Lambley: EN	Grassland & Heathland	1.54	2.8	38.75	10.08	3.84	711	*Quercus robor/petraea*	4.5	0.2	0.45	0.00	0.00	0.2	5.6	0.90	0.88	2.80	2.44	-1.9
19	Fressingfield	TM261759	Allison Collins: EN	Unimproved neutral grass	5.29	3.7	74.32	10.72	6.93	669	*Quercus robor/petraea*	5.4	0.7	0.58	20.70	2.31	-20	6.1	0	0	10.40	4.83	-10.4
20	Bedlington	TM173684	Allison Collins: EN	Unimproved neutral grass	7.43	3.8	96.55	11.04	8.75	684	*Quercus robor/petraea*	5.7	2.7	2.08	14.30	5.13	-11.6	5.3	0	0	6.00	2.00	-6.0
21	Borrowdale	NY240135	Bart Donato: EN	Upland oak woodland	0.19	2	40.10	28.64	1.40	2690	*Quercus robor/petraea*	4.3	8	5.42	0.00	0.00	8	5.9	0.22	0.44	0.11	0.33	0.11
22	Brown Moss	SJ563390	Chris Hogarth: EN	Woodland & heathland	3.95	2.7	57.91	8.96	6.46	803	*Quercus robor/petraea*	5.4	0	0.00	5.00	1.58	-5	5.8	0	0	9.90	3.28	-9.9
23	Llynclys	SJ273237	Chris Hogarth: EN	Grassland, scrub & wood	1.78	1.8	34.50	8	4.31	930	*Betula pubescens*	4.7	0	0.00	0.20	0.45	-0.2	5.6	0	0	1.10	1.45	-1.1
24	Wytham Wood	SP452083	Ron Porley: EN	Wood pasture	1.13	3.6	33.09	9.12	3.63	715	*Quercus robor/petraea*	4.0	0	0.00	0.00	0.00	-	5.4	0.75	0.89	1.63	1.41	-0.88
25	Lullington Heath	TQ538016	Malcolm Emery: EN	Chalk heath & chalk grassland	0.9	3.3	27.84	11.04	2.52	877	*Crateagus monogyna*	4.2	0	0.00	0.00	0.00	-	5.2	15.50	4.14	1.67	1.51	13.83
26	Lough Navar	H074545	Melina McMullan: EHS	Oak woodland & blanket bog	0.47	0.6	11.48	9.76	1.18	1364	*Betula pubescens*	4.5	17.6	8.05	0.00	0.00	17.6	5.3	0.14	0.38	0.57	1.51	-0.43
27	Glenmore Wood	H654608	Melina McMullan: EHS	Mature acid oak woodland	2.28	0.7	32.49	8.8	3.69	970	*Quercus robor/petraea*	4.5	0	0.00	0.00	0.00	-	6	0	0	2.67	3.50	-2.7
28	Caldanagh Bog	D022205	Melina McMullan: EHS	Lowland raised bog	2.58	1.5	36.33	9.6	3.78	1056	*Fraxinus excelsior*	5.4	0.00	0.00	4.80	6.57	-4.8	6.1	0	0	9.50	4.45	-9.5
29	Castle Enigan	J121322	Melina McMullan: EHS	Lowland Fen	4.09	2	53.53	10.08	5.31	1044	*Crateagus monogyna*	4.9	0.00	0.00	0.00	0.00	-	6.4	0	0	10.10	3.60	-10.1
30	Orritor	H768782	Melina McMullan: EHS	Oak woodland	6.45	3.9	72.98	9.92	7.36	1183	*Fraxinus excelsior*	5.7	0.00	0.00	0.00	0.00	-	6.4	0	0	6.00	3.46	-6.0
31	Redgrave & Lopham Fen	TM050797	Andrew Excell: SWT	Fenland	2.28	3.7	45.30	10.4	4.36	677	-	-	-	-	-	-	-	-	-	-	-	-	-
32	Yarner Wood	SX786789	Pat Wolseley: NHM	Ancient oak woodland	0.65	1.1	31.22	18.24	1.71	1635	*Quercus robor/petraea*	4.3	11.50	2.38	0.00	0.00	11.5	4.8	11.80	6.92	1.33	1.03	10.5

Notes: 1, Mean measured NH_3 concentration for the period 2000-2004. 2, Site-based and interpolated measured values for 2001-2003. 3, Modelled values for 2001-2003, according to the method of Smith *et al.* (2000).

Supplementary Section 6: Effects of controlled ammonia exposure on peatland vegetation

Experimental Site

The experiment is carried out at Whim bog in the Scottish Borders (3° 16' W, 55° 46' N) on 3-6 m of peat. Mean temperatures of the air and soil (at 10 cm depth) were 7.9 °C and 7.6 °C respectively with mean annual rainfall (2011 to 2016) of 1141 mm (range: 734-1486 mm). The peat is very acidic, with pH 3.4 (3.27-3.91 in water). The vegetation is mainly classified as a *Calluna vulgaris* - *Eriophorum vaginatum* blanket mire community (M19 in the UK National Vegetation Classification). Prior to treatment and in controls, the replicate plots represent highly variable mosaics containing *Calluna vulgaris* and *Sphagnum capillifolium* hummocks, with hollows containing *S. fallax* and *S. papillosum*. Other common species include *Erica tetralix* and the mosses *Hypnum jutlandicum* and *Pleurozium schreberi*.

Experimental Treatments

Nitrogen is applied to the site using two different treatment systems, one for dry deposition of NH_3 gas, and one for wet deposition of NH_4^+ and NO_3^- in solution. Treatments commenced in June 2002 and continue through the year, except during winter (i.e., freezing conditions prevent operation of the wet release system). NH_3 deposition is manipulated using a free-air release system, supplied from a cylinder of liquid NH_3, diluted with ambient air and released from a perforated 10 m pipe, 1 m above ground. NH_3 is released only when wind direction was in the south-west (from 180° to 215°) and wind speed exceeded 2.5 m s^{-1} (Leith *et al.*, 2004). Ammonia concentrations are measured 0.1 m above the vegetation using ALPHA samples (Tang *et al.*, 2001) at 8 distances downwind of the source, as well as background. Ammonia dry deposition is calculated from the concentration measurements using the method of Cape *et al.* (2008). This bespoke method applies a canopy resistance (R_c) based inferential modelling approach using monitored NH_3 concentrations at 0.1 m above the canopy. The main uncertainty is the extent to which R_c values change as a function of NH_3 concentration. Therefore a specific concentration-dependent function of R_c is used, derived from detailed chamber measurements for this habitat by Jones *et al.* (2007).

Wet deposition of NH_4^+ and NO_3^- is increased in replicated mesocosms in a randomised block design, using a water sprayer system using collected rainwater to dilute solutions of NH_4Cl or $NaNO_3$, to cover each plot (12.8 m^2) uniformly. In addition to a background level of 8 kg N ha^{-1} y^{-1}, three treatment levels are applied, aiming to provide total (background + treatment) nitrogen of 16, 32 and 64 kg N ha^{-1} y^{-1}.

The dry ammonia transect is 10 m wide by 60 m long, which was designed to allow for multiple samples of individual species at each concentration along the transect. This provides a sufficiently large sampling pool to assess changes in the species within the habitat from the line source, allowing variation to be considered with distance from the NH_3 source, as well as variation between permanent replicated quadrats at each sampling distance, exposed to the same NH_3 levels. Use of multiple NH_3 line sources of this scale would increase the sampling pool, but would not be feasible due to the environment impact at this experimental study site, and because of the multiplication of sampling effort and resources that would be required.

Conversely, use of smaller transects would not provide the real world scenario that is created by the Whim experiment.

The major changes in species along the NH_3 transect demonstrate the successful design, while increasing the sampling pool further would be unlikely to improve relationships substantially. If substantial extra financing were available, rather than increasing replication, it would be considered a priority to invest in additional experiments of the type developed at Whim Bog, *for other ecosystem types*, to address the extent to which the observed differential responses between NH_3, NH_4^+ and NO_3^- are also found in other habitats.

The NH_4^+ and NO_3^- wet treatments have four 12.5m^2 plots for each N level, and were also designed in size to allow for multiple samples of individual species within each plot. This provides a high level of replication for each species, across a number of replicated plots.

Effects of the treatments on peat carbon and nitrogen interactions have been reported elsewhere by Kivimäki *et al.* (2013) and Sheppard *et al.* (2013).

Plant survey, leaf surface pH and physiological measurements

Species survey

Plant species composition has been surveyed in all plots over the course of the experiment, usually every two years. In each experimental plot, three permanent quadrats (40 x 40 cm) were established. These were sub-divided into 16 sub-quadrats (10 x 10 cm). At each survey, the percent cover of all species is recorded at the sub-quadrat level. For each species, the 16 sub-quadrat values are averaged to give a mean cover for each quadrat. For further details of the site, treatments and plant survey analysis, see Sheppard *et al.* (2014) and Levy *et al.* (2019).

Leaf surface pH

Surface pH of *Sphagnum capillifolium* and *Cladonia portentosa* were scored by modifying the methodology of Costa *et al.* (2005). Intensive measurements were done *in situ* at the CEH Whim experimental field site with a pH meter (Horiba LAQUAact-pH 110) having a flat-bottom pH electrode from June to September 2018, supplemented by subsequent measurements in 2019. The surface of these *Sphagnum* and *Cladonia* species were moistened with deionized (DI) water and were allowed to wet. After 2-3 minutes, a drop of DI water (0.1 ml) was pipetted on the surface of *Sphagnum* and *Cladonia* and left for 10 seconds prior to pH measurement; the measurement was then repeated to ensure a stable reading. The flat tip of the pH electrode was put on the individual *Sphagnum* and *Cladonia* plants held by the tweezer from beneath, and the pH was recorded on the screen of the digital pH meter. The temperature was set at MTC (20 °C). Before the measurements, the pH meter electrode was immersed in KCl (3.33 mol L^{-1}) solution and then calibrated with pH 4 and pH 7 solutions.

Physiological indicator measurements

An indication of plant photosynthetic functioning was provided by measuring fluorescence of two lichen species, transplanted into the NH_4^+, NO_3^- and NH_3 plots: *Evernia prunastri* (representative of an acidophyte species) and *Xanthoria parietina* (representative of a nitrophyte species). Fluorescence measurements using a Plant Efficiency Analyzer (Handy

PEA, Hansatech Ltd, Norfolk, UK) were carried out at three periods allowing up to 6 months field exposure of transplants (Munzi *et al.*, 2014). Fully hydrated samples were dark adapted for 10 min, then illuminated for 1 s with a saturating (3000 μmol m^{-2} s^{-1}) red-light pulse and fluorescence emission was recorded for 1 s. The parameter F_v/F_m, the maximum quantum yield of primary photochemistry, was used as a proxy of the intrinsic efficiency of photosystem II (PSII) and therefore as a vitality index for the samples, which corresponds to:

$$F_v/F_m = (F_m - F_0)/F_m \quad \text{[E3]}$$

where F_0 and F_m are the minimum (before the light pulse, when all the reaction centres of PSII are open) and maximum chlorophyll *a* fluorescence (after the light pulse, when all the reaction centres are closed) emission, and F_v is the variable fluorescence, the difference between the maximum and the minimum. Optimal values for healthy lichens and mosses vary around 0.6 and 0.8.

Supplementary Results

Plant species cover

Data are examined here for five common species. These were the only species that frequently occurred with more than 5 % cover: *Calluna vulgaris*, *Sphagnum capillifolium*, *Cladonia portentosa*, *Hypnum jutlandicum* and *Pleurozium schreberi*. In addition, *Eriophorum vaginatum* was abundant with over 5% cover, as further analysed by Levy *et al.* (2019). This plant is not considered here, since it was found to benefit from gaseous NH_3 exposure relative to the more sensitive plant species. The data reported by Levy *et al.* (for 2002-2016) are here extended for plant cover sampling up to and including 2019.

The results of the plant cover sampling are illustrated in Figure S5a-c for *Calluna vulgaris*, *S. capillifolium* and *C. portentosa*, respectively. It is immediately obvious that the effects of NH_3 on percentage cover are much larger and occur more rapidly than for wet deposited NH_4^+ and NO_3^-. Of the three species shown, *C. portentosa* is found to be the most sensitive and *C. vulgaris* the least sensitive.

In order to compare the outcomes for the five key species, the data for each treatment (by nitrogen form, N application rate and species) are here used to estimate the time taken to reduce cover by 50% compared with the start of the experiment (ET_{50}). Linear interpolation was used, except where the data were obviously non-linear, where simple decay curves were fitted (as shown in Figure S5).

The Eradication Dose 50 (ED_{50}) is defined as the annual nitrogen deposition multiplied by the time taken to reduce cover by 50% compared with the start of the experiment (kg N ha^{-1}). In Figure 6 (main text) the calculated results for ED_{50} are plotted versus the annual N deposition in the experimental treatments in order to visualize the outcomes, and see to what extent the ED_{50} may itself depend on the N deposition rate. A summary of the ET_{50} and ED_{50} results and the calculations underpinning Figure 7 is given in Table S7.

Statistical comparison was based on reciprocal values of ED_{50}, which helped normalize the data compared with the skew distribution of ED_{50} values (Figure 6). This also allowed the reciprocal value of ED_{50} to be set at 0 for treatments where plant cover was stable or

increased over time. Making this correction would have tended to underestimate the differences between treatments shown in Figure 7.

Since the experiment was established in 2002, background nitrogen deposition has decreased by around 20% (Levy *et al.*, 2020) and it is possible to calculate the likely effect on the estimation of total N dose of the 18 year exposure period of the experiment. Based on total N deposition in 2018 being 20% less than in 2001, then the deposition rates for 2018 would be 6.4 (background), 14.4, 30.4 and 62.4 kg^{-1} y^{-1}. Since the background N deposition rate decreased approximately linearly through the period, then the total accumulated N doses of the 18 year period can be estimated as being slightly smaller than indicated by the nominal annual rates of 8 (background), 16, 32, 64 kg N ha^{-1} y^{-1}, by 10% (background), 5%, 3% and 1%, respectively. This indicates only a minor effect on the calculated ED_{50} values shown in Figure 6 and Table S7. A further change during the life of the experiment is that background precipitation is no longer as acidic as it was during 2001 (Figure 2C).

Interactions with leaf surface pH

Wet deposition of NH_4^+ and NO_3^- is also associated with potential acidity and alkalinity interactions. Hence plant uptake of deposited NH_4^+ (to become R-NH_2 in organic compounds) releases H^+, with additional acidity released if NH_4^+ is nitrified to NO_3^-, which may then be leached accompanied by loss of base cations (Sutton *et al.*, 1993). By contrast, reduction of NO_3^- to NH_4^+ consumes H^+, although this effect can be partly or fully offset if this is then converted to organic R-NH_2 forms. In an acidic peatland system, with limited nitrification expected, a net acidifying effect of NH_4^+ may be expected (to the extent that this is taken up by plants), while NO_3^- has potential for a weak alkaline effect. As these effects are dependent on plant uptake and also result from low aqueous N concentrations (compared with high ammoniacal concentrations on plant surfaces from exposure to gaseous NH_3), then much smaller pH effects are expected compared with gaseous NH_3. Comparison of the surface pH of *Sphagnum capillifolium* between wet deposition NH_4^+ and NO_3^- treatments showed no significant differences (cf. Figure S8). By contrast, limited measurements of lichens growing on wooden walkways ('board-walks') adjacent to some plots did show pH differences (data not shown), which may be examined in a future publication.

Differences in physiological response to N forms

The photosynthetic indicator F_v/F_m showed that both the transplanted lichen species were more affected by exposure to gaseous NH_3 than to wet deposited NH_4^+ and NO_3^- (Figure S6). In the case of the nitrophyte species *Xanthoria parietina*, there was no apparent effect of either wet deposited NH_4^+ or NO_3^- on F_v/F_m, whereas for the acidophyte species *Evernia prunastri*, the effect of NH_4^+ was intermediate between NO_3^- and gaseous NH_3.

Table S7: Estimated Eradication Time 50 (ET_{50}) to halve species cover compared with the start of the experiment (**see Supplementary Figure S5a**) and the calculated Eradication Dose 50 (ED_{50}). Notes: ND, not determined where cover was stable or increased over time. The reciprocals of these values were set to zero ([a]).

		**ET_{50} : time to reduce cover by 50% compared with initial conditions (years)**						
**N form applied**	**N rate (kg N ha^{-1} y^{-1})**	***Calluna vulgaris***	***Sphagnum capillifolium***	***Cladonia portentosa***	***Pleurozium schreberi***	***Hypnum jutlandicum***		
**NH_3**	16	37.4	31	2.6	9.4	26.6		
"	32	3.8	1.6	0.6	1.5	4.9		
"	64	1.9	1.8	1.3	2.4	4.2		
**NH_4^+**	16	ND	ND	7.3	8.2	9.3		
"	32	32.8	9.9	8.7	8.7	48.6		
"	64	25.2	3.4	2.9	1.3	ND		
**NO_3^-**	16	ND	53.2	14.5	12.4	ND		
"	32	ND	16.9	8.8	7.8	ND		
"	64	19.4	16.9	2.1	3.3	ND		
		**ED_{50} dose to reduce cover by 50% compared with initial conditions (kg N ha^{-1})**						
**N form applied**	**N rate (kg N ha^{-1} y^{-1})**	***Calluna vulgaris***	***Sphagnum capillifolium***	***Cladonia portentosa***	***Pleurozium schreberi***	***Hypnum jutlandicum***		
**NH_3**	16	598	496	42	150	426		
"	32	122	51	19	48	157		
"	64	122	115	83	154	269		
**NH_4^+**	16	ND	ND	117	131	149		
"	32	1050	317	278	278	1555		
"	64	1613	218	186	83	ND		
**NO_3^-**	16	ND	851	232	198	ND		
"	32	ND	541	282	250	ND		
"	64	1242	1082	134	211	ND		
		**Reciprocal of ED_{50} (ha kg^{-1} N)**					**ED_{50} Species mean (kg N ha^{-1})**	
**N form applied**	**N rate (kg N ha^{-1} y^{-1})**	***Calluna vulgaris***	***Sphagnum capillifolium***	***Cladonia portentosa***	***Pleurozium schreberi***	***Hypnum jutlandicum***	**by N level**	**by N form**
**NH_3**	16	0.00167	0.00202	0.02404	0.00665	0.00235	136	82
"	32	0.00822	0.01953	0.05208	0.02083	0.00638	47	
"	64	0.00822	0.00868	0.01202	0.00651	0.00372	128	
**NH_4^+**	16	0 [a]	0 [a]	0.00856	0.00762	0.00672	175	244
"	32	0.00095	0.00316	0.00359	0.00359	0.00064	419	
"	64	0.00062	0.00460	0.00539	0.01202	0 [a]	221	
**NO_3^-**	16	0 [a]	0.00118	0.00431	0.00504	0 [a]	475	443
"	32	0 [a]	0.00185	0.00355	0.00401	0 [a]	532	
"	64	0.000805	0.00092	0.00744	0.00474	0 [a]	360	
		**ED_{50} Mean values for each N form and species (kg N ha^{-1})**						
**NH_3**	All data	166	99	34	88	241		
**NH_4^+**	All data	1911	387	171	129	408		
**NO_3^-**	All data	3727	759	196	218	-		

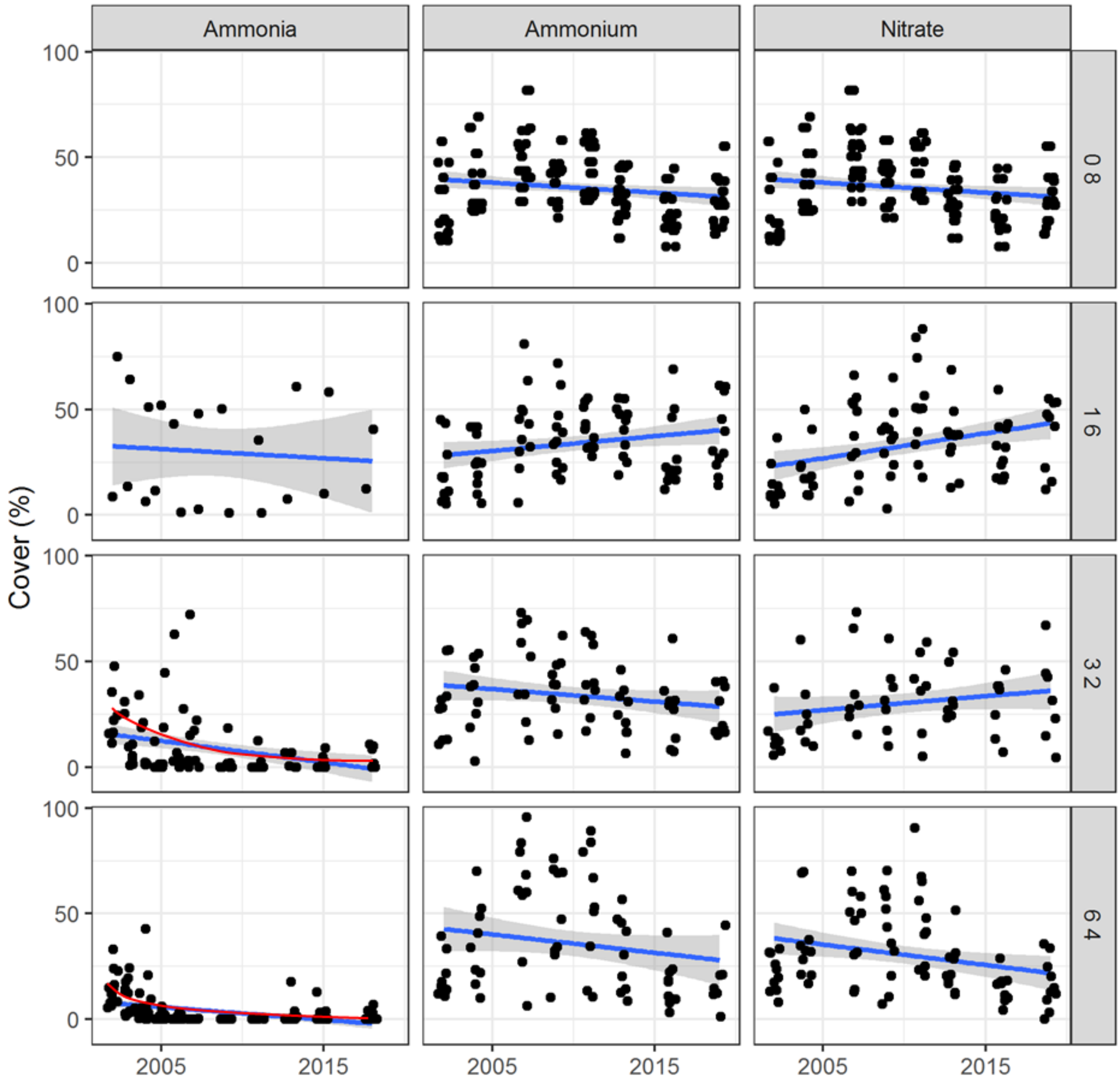

Figure S5a: Response of *Calluna vulgaris* cover to addition of gaseous NH_3 and wet deposition of NH_4^+ and NO_3^- at rates of 8 (control), 16, 32 and 64 kg N ha^{-1} y^{-1} (averages of replicated plots).

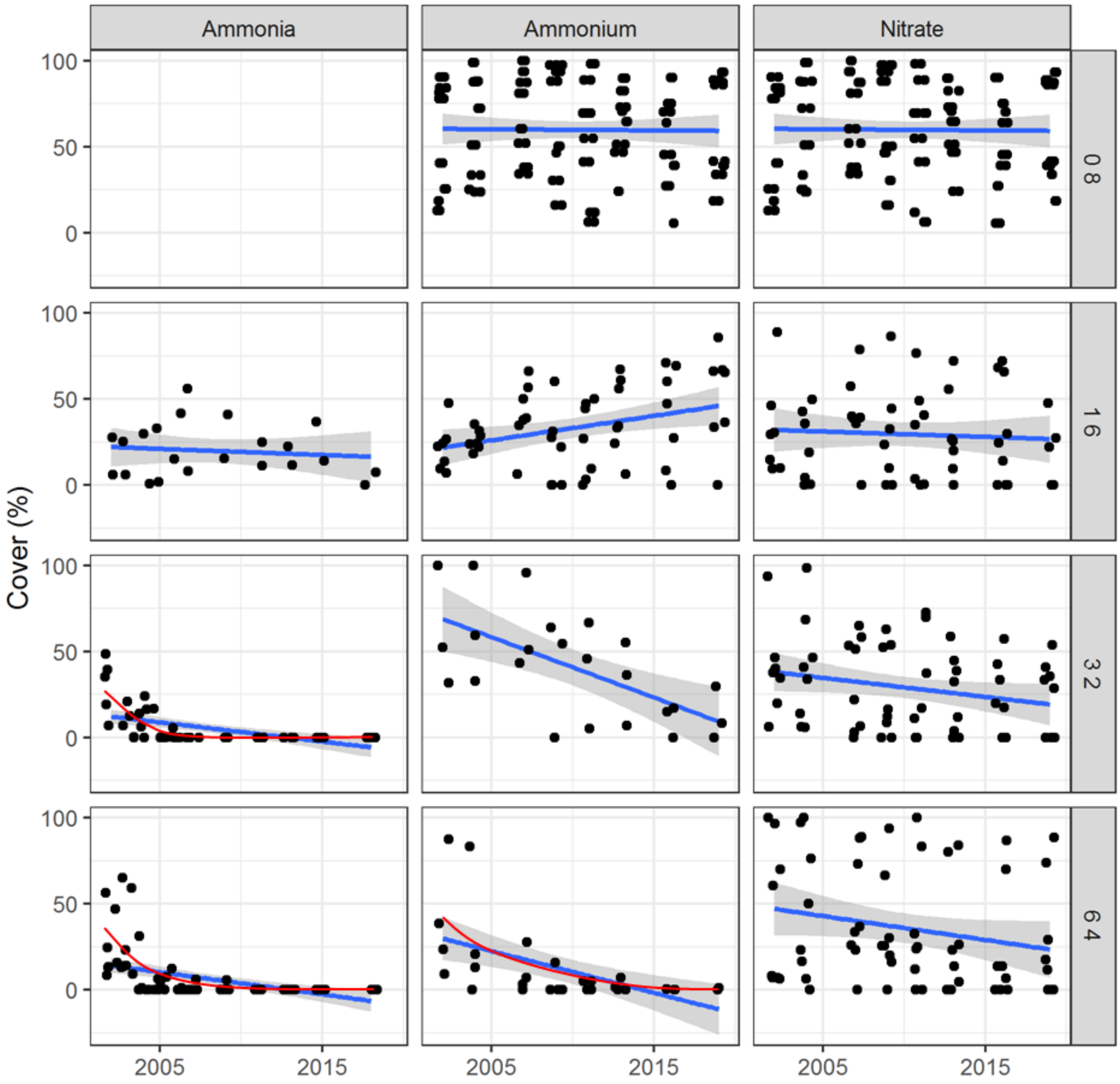

Figure S5b: Response of *Sphagnum capillifolium* cover to addition of gaseous NH_3 and wet deposition of NH_4^+ and NO_3^- at rates of 8 (control), 16, 32 and 64 kg N ha^{-1} y^{-1} (averages of replicated plots).

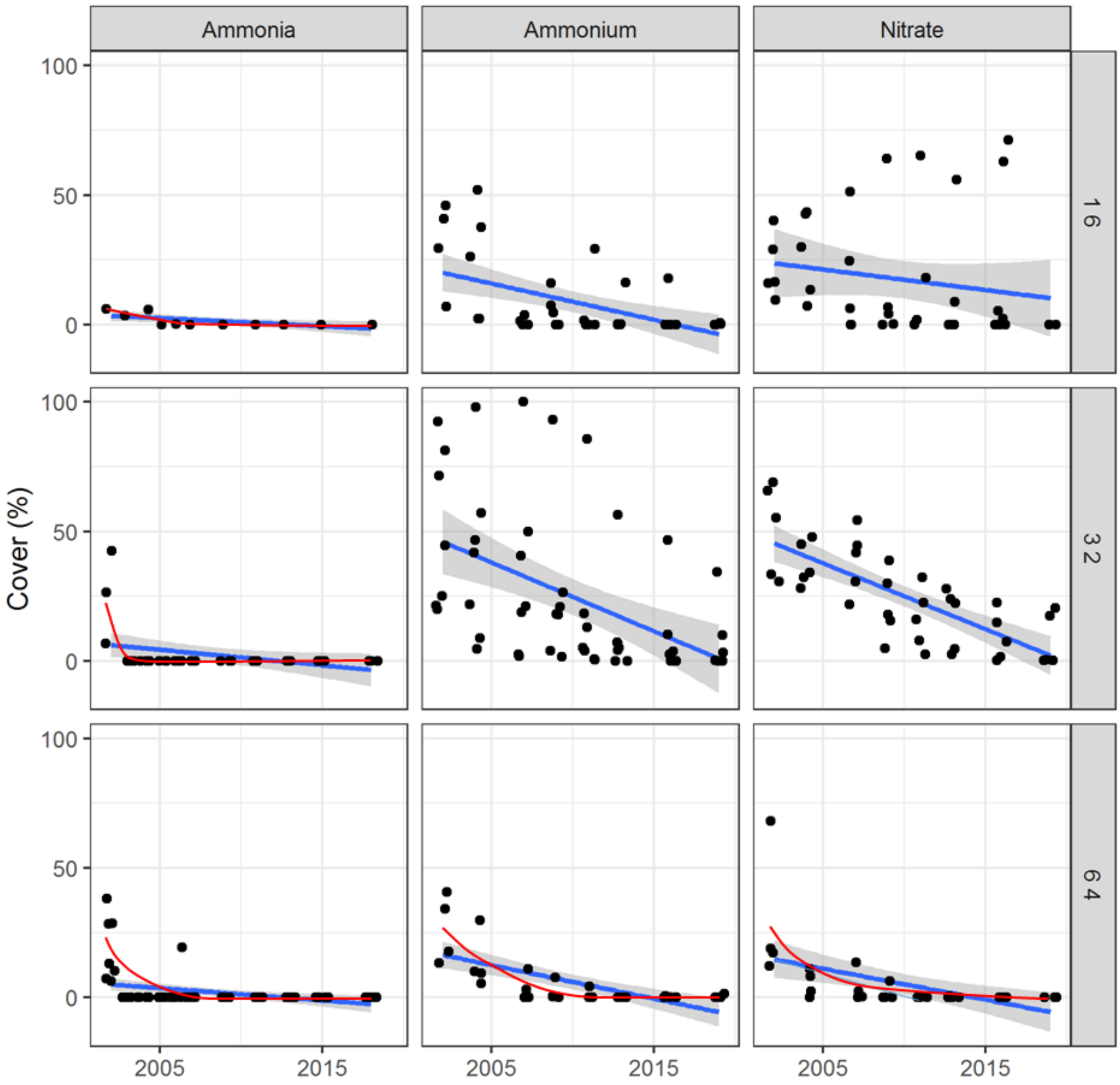

Figure S5c: Response of *Cladonia portentosa* cover to addition of gaseous NH_3 and wet deposition of NH_4^+ and NO_3^- at rates of 8 (control), 16, 32 and 64 kg N ha^{-1} y^{-1} (averages of replicated plots).

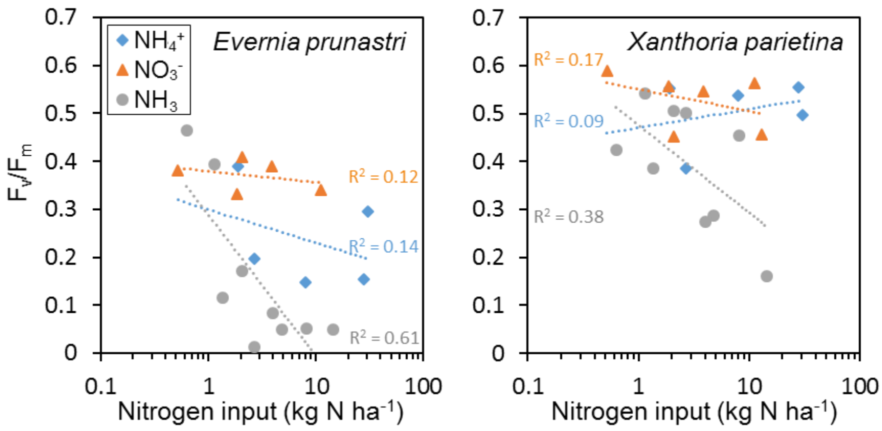

Figure S6: Estimation of the photosynthetic indicator F_v/F_m in transplants of two lichen species, exposed for up to 6 months at Whim Bog to wet deposition of NH_4^+ or NO_3^- and to dry deposition of NH_3. Total deposition is shown based on transplant exposure-time, considering the timing of preceding wet and dry N release periods. Least square regressions were fitted as $F_v/F_m = a \ln(\text{N input}) + b$.

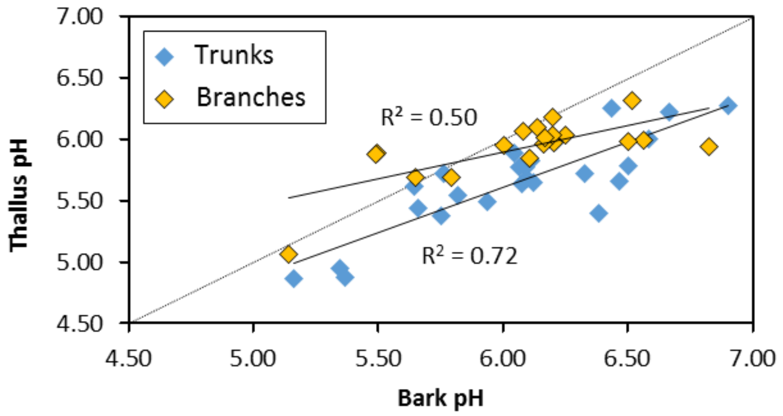

Figure S7: Relationship between bark pH and lichen thallus pH shown for *Xanthoria parietina* occurring on trees in a region of high NH_3 concentrations in the Po Valley, Italy. Data for trunks are for *Quercus cerris*, *Q. robur*, *Juglans regia*, *Tilia cfr. platyphyllos* and *Aesculus hippocastanum* (n=25). Data for branches are for *Q. cerris*, *Q. robur* and *J. regia* (n=20).

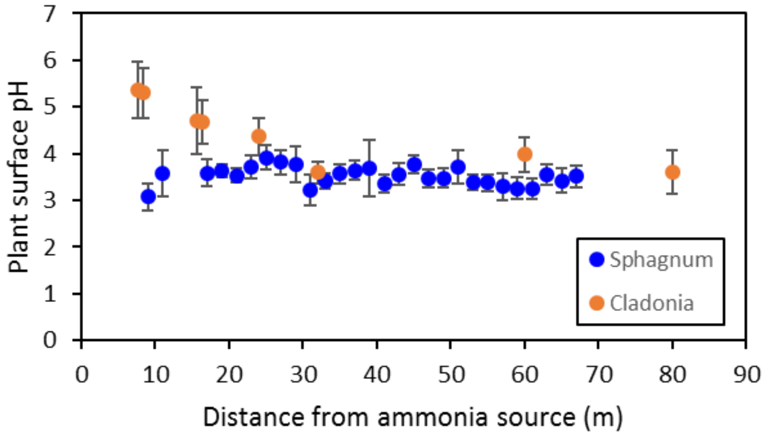

A.

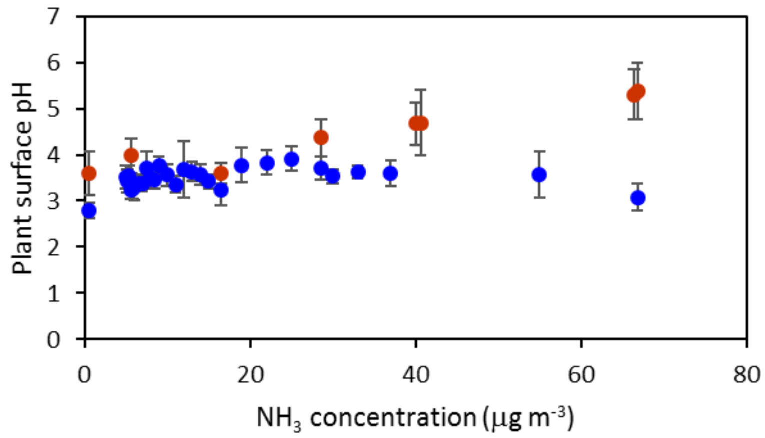

B.

Figure S8: Surface pH of *Sphagnum capillifolium* and *Cladonia portentosa* measured along the NH_3 transect at Whim Bog, shown in relation to distance from source (A) and NH_3 concentrations (B). For *Sphagnum*, 10 measurements were made per location on *in situ* plants (July 2018). For *Cladonia*, specimens were transplanted into the transect on 16 August 2018, with pH measurements on 6, 12 September 2018, 11 October 2018 and 24 September 2019. Values for *Cladonia* are means and standard deviations of the four sampling periods (based on triplicate measurements at each distance).

Supplementary References

For other references see main text.

Abt T. (2011) The Book of Pictures *Muṣḥaf aṣ-ṣuwar* by Zosimos of Panopolis (ed.) trans. S. Fuad and T. Abt. Zurich: Living Human Heritage Publications.

Abt T. (2016) The Book of the Keys of the Work (*Kitāb Mafātīḥ aṣ-ṣan'a*) by Zosimos of Panopolis (ed.) (trans. Fuad S, Abt T). Living Human Heritage Publications, Zurich

Anonymous (1819, 1824) *The Asiatic Journal* (October 1819; January 1825) https://books.google.co.uk/books?id=JfNdAAAAcAAJ&dq=%22sal+ammoniac%22+price+1800&source=gbs_navlinks_s (Accessed: 05 April 2019)

Anonymous (1853) On the manufacture of ammonia and ammoniacal salts. *Journal of Pharmacy* **2**, 263.

Asta J., Erhardt W., Ferretti M., Fornasier F., Kirschbaum U., Nimis P.L., Purvis O.W., Pirintsos S., Scheidegger C., Van Haluwyn C. & Wirth V. (2000) Mapping lichen diversity as an indicator of environmental quality. In Nimis, P.L, Scheidegger, C. & Wolseley, P.A. (eds.) *Monitoring with Lichens – Monitoring Lichens*. NATO Science Series, Kluwer Academic Publishers, Dordrecht: pp 273-280.

Berthelot M. and Ruelle C.E. (1888) *Collection des Anciens Alchimistes Grecs*. Paris: George Steinheil.

Cape J.N., Jones, M.R., Leith, I.D., Sheppard, van Dijk, N., Sutton, M.A. & Fowler, D. (2008) Estimate of annual NH_3 dry deposition to a fumigated ombrotrophic bog using concentration-dependent deposition velocities. *Atmospheric Environment* **42**, 6637-6646.

Clow A. & Clow N.L. (1952) *The Chemical Revolution. A Contribution to Social Technology*. Reprinted 1970. Books for Libraries Press, Freeport, New York.

Colinet A. (2000) Le Travail des quatre éléments ou lorsqu'un alchimiste byzantin s'inspire de Jabir. In: *Occident et Proche-Orient: Contacts scientifiques au temps des Croisades*. (eds.: I. Draelants, A. Tihon & B. van den Abeele), pp 165-190. Turnhout, Belgium: Brepols.

Commissioner of Agriculture (1870) Report of the Commissioner of Agriculture for the year 1869. Government Printing Office, Washington. https://books.google.co.uk/books?id=R2BTAAAAcAAJ&source=gbs_navlinks_s (Accessed: 05 April 2019)

Costa N.V. da, Martins D., Rodella R.A. & de Camargo da Costa, L.D.N. (2005) Droplet deposition during spray and leaf pH in aquatic weed control. *Scientia Agricola* **62**(3), 227-234.

Defra (2019) Defra National Statistics Release: Air quality statistics in the UK 1987 to 2018 (25 April 2019). https://assets.publishing.service.gov.uk/government/uploads/system/uploads/attachment_data/file/796887/Air_Quality_Statistics_in_the_UK_1987_to_2018.pdf (Accessed: 05 April 2019)

Goldschmidt G. (1923) *Heliodori carmina quattuor ad fidem codicis Casselani*. Giessen: Töpelmann.

Hallum B. (2008) Zosimus Arabus: the reception of Zosimos of Panopolis in the Arabic/Islamic world. PhD dissertartion. Warburg Institute, University of London.

Hallum B. (2009) The *Tome of Images*: an Arabic Compilation of Texts by Zosimos of Panopolis and a Source of the *Turba Philosophorum*. *Ambix* **56**, 76-88.

Hansen V. (2005) The Impact of the Silk Road Trade on a Local Community: The Turfan Oasis, 500-800. In : *Les Sogdiens en Chine*. (eds. De La Vaissière, É. & Trombert, É.), pp 283-310. Paris: École Française d'Extrême-Orient (Etudes thématiques, 17).

Jones M.R., Leith I.D., Fowler D., Raven J.A., Sutton M.A., Nemitz E., Cape J.N., Sheppard L.J., Smith R.I.and Theobald M.R. (2007). Concentration-dependent NH_3 deposition processes for mixed

moorland semi-natural vegetation. *Atmospheric Environment* **41**, 2049–2060. doi:10.1016/j.atmosenv.2006.11.003.

Kivimäki S.K., Sheppard L.J., Leith I.D., Grace J. (2013) Long-term enhanced nitrogen deposition increases ecosystem respiration and carbon loss from a Sphagnum bog in the Scottish Borders**.** *Environmental and Experimental Botany* **90**, 53-61

Kunsman C.H. (1940) Sources and available supplies of commercial fertilizers. Misc. Publication 400. Washington D.C.:United States Department of Agriculture. https://books.google.co.uk/books?id=yLRG7pt8rTsC&source=gbs_navlinks_s (Accessed: 05 April 2019)

Leith I.D., van Dijk N., Pitcairn C.E.R., Wolseley P.A., Whitfield C.P. & Sutton M.A. (2005) Biomonitoring methods for assessing the impacts of nitrogen pollution: refinement and testing (eds). Report 386. Joint Nature Conservation Committee, Peterborough, 290 pp. www.jncc.gov.uk/default.aspx?page=3886

Leith I.D., Sheppard L.J., Fowler D., Cape J.N., Jones M.R., Crossley A., Hargreaves K.J., Tang Y.S., Theobald M. & Sutton M.A. (2004) Quantifying dry NH_3 deposition to an ombrotrophic bog from an automated NH_3 field release system. *Water, Air and Soil Pollution: Focus* **4**, 207-218.

Levy P.E., Smith R.I., Tang Y.S., Stedman J.R. (2020). Deposition of nitrogen, sulphur and base cations to the UK (1986-2012). NERC Environmental Information Data Centre. (Dataset). https://doi.org/10.5285/8e7644fe-9f17-4fc3-8e4e-8b10a42d5d50 (Accessed: 24 July 2020).

Munzi S., Cruz C., Branquinho C., Pinho P., Leith I.D. & Sheppard L.J. (2014) Can ammonia tolerance amongst lichen functional groups be explained by physiological responses? *Environmental Pollution* **187**, 206-209.

Papathanassiou M.K. (2017) *Stephanos von Alexandreia und sein alchemistisches Werk.* Athen.

Pitcairn C.E.R., Skiba U.M., Sutton M.A., Fowler D., Munro R. & V.K. Kennedy (2002) Defining the spatial impacts of poultry farm ammonia emissions on species composition of adjacent woodland groundflora using Ellenberg indicators, nitrous oxide and nitric oxide and foliar nitrogen as marker variables. *Environmental Pollution* **119**, 9-21.

Pitcairn C., Fowler D., Leith I., Sheppard L., Tang S., Sutton M.A. & Famulari D. (2006) Diagnostic indicators of elevated nitrogen deposition. *Environmental Pollution* **144**, 941-950.

Read J. (1938) Alchemy under James IV of Scotland. *Ambix* **2**, 60-67.

Ruska J. (1923) Sal ammoniacus, Nušādir und Salmiak. *Sitzungsberichte der Heidelberger Akademie der Wissenschaften (Philosophisch-historiche Klasse)* (May 1923). C. Winter, Heidelberg. 23 pp.

Sheppard L.J., Leith I.D., Leeson S.R., van Dijk N., Field C., Levy P. (2013) Fate of N in a peatland, Whim bog: immobilisation in the vegetation and peat, leakage into pore water and losses as N_2O depend on the form of N. *Biogeosciences* **10**, 149-160. https://doi.org/10.5194/bg-10-149-2013

Sheppard L.J., Leith I.D., Mizunuma T., Leeson S., Kivimaki S., Cape J.N., Dijk N., Leaver D., Sutton M.A., Fowler D., Berg L.J.L, Crossley A., Field C. & Smart S. (2014) Inertia in an ombrotrophic bog ecosystem in response to 9 years' realistic perturbation by wet deposition of nitrogen, separated by form. *Global Change Biology* **20**, 566-580.

Simpson D., Benedictow A., Berge H., Bergström R., Emberson L.D., Fagerli H., Flechard C.R., Hayman G.D., Gauss M., Jonson J.E., Jenkin M.E., Nyíri A., Richter C., Semeena V.S., Tsyro S., Tuovinen J.P., Valdebenito Á., Wind P. (2012) The EMEP MSC-W chemical transport model - technical description, *Atmos. Chem. Phys.* **12**, 7825-7865, doi:10.5194/acp-12-7825-2012.

Skamarock W.C., Klemp J.B., Dudhia J., Gill D.O., Liu Z., Berner J., Wang W., Powers J.G., Duda M.G., Barker D.M., Huang X.-Y. (2019) A description of the Advanced Research WRF Version 4. NCAR Tech. Note NCAR/TN-556+STR, 145 pp. doi:10.5065/1dfh-6p97

Smith R.I., Fowler D., Sutton M.A., Flechard C. & Coyle M. (2000) Regional estimation of pollutant gas deposition in the UK: model description, sensitivity analyses and outputs. *Atmos. Environ.* **34**, 3757-3777.

Stapleton H.E. (1953) The antiquity of alchemy. *Ambix* **5**, 1-43.

Stapleton H.E. & Azo R.F. (1910) An alchemical compilation of the thirteenth century, A.D. *Memoirs of the Asiatic Society of Bengal* **3**, 57-94.

Sutton M.A., Pitcairn C.E.R. & Fowler D. (1993) The exchange of ammonia between the atmosphere and plant communities. *Advances in Ecological Research* **24**, 301-393.

Sutton M.A., Pitcairn C.E.R. & Whitfield C.P. (2004) (eds.) *Bio-indicator and Bio-monitoring methods for Assessing the Effects of Atmospheric Nitrogen on Statutory Nature Conservation Sites.* JNCC Report No. 356. JNCC, Peterborough.

Tang Y.S., Cape J.N. & Sutton M.A. (2001) Development and types of passive samplers for NH_3 and NO_x. *TheScientificWorld* **1**, 513-529.

Tang, Chanyu (1992) editor. *Tulufan chutu wenshu*. Volume 1. Beijing: Wenwu Press. [“Documents excavated at Turfan”, in Chinese].

US Department of Commerce (1935), Statistical Abstract of the United States (number 57). https://books.google.co.uk/books?id=yCNUR78TQoYC&source=gbs_navlinks_s (Accessed: 05 April 2019)

US Department of Labor, Bureau of Labor Statistics (1923) Wholesale prices 1890 to 1922. Washington: Government Printing Office. https://books.google.co.uk/books?id=D54mAQAAIAAJ&source=gbs_navlinks_s (Accessed: 05 April 2019)

Van Herk C.M. (2000) Epiphytes on wayside trees as an indicator of eutrophication in the Netherlands. In: Nimis, P.L, Scheidegger, C. & Wolseley, P.A. (eds.) *Monitoring with Lichens – Monitoring Lichens.* pp 285-290. NATO Science Series, Kluwer Academic Publishers, Dordrecht.

VDI (1995) Messung von Immissionswirkungen: Ermittlung und Beurteilung phytoxischer Wirkungen von Immisssion mit Flechten – Flechtenkartierung zur Ermittlung des Luftgütewertes (LGW), *VDI- Richtlinie 3799*, Blatt 1, Berlin.

Vieno, M., Heal, M. R., Williams, M. L., Carnell, E. J., Nemitz, E., Stedman, J. R., and Reis, S.: The sensitivities of emissions reductions for the mitigation of UK $PM_{2.5}$, *Atmos. Chem. Phys.*, 16, 265-276, 10.5194/acp-16-265-2016, 2016.

Wirth V. (1992) Zeigerwerte von Flechten. *Scripta Botanica* **18**, 215-237.

Wolseley P.A., Leith I.D., van Dijk N. & Sutton M.A. (2005) Extensive UK scale study: Comparison of expert and non-expert sampling. Chapter 12, In: *Biomonitoring methods for assessing the impacts of nitrogen pollution: refinement and testing* (eds: Leith I.D., van Dijk N., Pitcairn C.E.R., Wolseley P.A., Whitfield C.P. & Sutton M.A.) pp 183-188, Report 386. Joint Nature Conservation Committee, Peterborough, 290 pp.

Wolseley P.A., Leith I.D., van Dijk N. & Sutton, M.A. (2009). Macrolichens on twigs and trunks as indicators of ammonia concentrations across the UK – A practical method. In: *Atmospheric Ammonia.* (eds: Sutton M.A., Reis S. & Baker S.M.H.), pp. 101-109. Springer Science & Business Media, B.V.

Appendix A:
A Greek list of alchemical names for aqueous ammonia

The Greek 'Work of the Four Elements' appears to be a translation (c. 850 to 1478) of an Arabic treatise of Jābir, to which the Greek editor has added several groups of Greek synonyms (see Supplementary Section 3; Colinet, 2000). It is relevant for the history of ammonia as it shows: a) distillation and processing of ammoniacal solutions, and b) that the editor identified these solutions according to names characteristic of the earliest known Greek alchemy (from around a millennium earlier). Comparison with other texts (e.g., attributed to Hermes, Democritus, Zosimus, Synesius) suggests that it would not have been acceptable for the earliest recognized Greek alchemists to have written with such clarity. With increasing openness in Islamic alchemy, the Greek editor was able to translate what had already been written, then adding the synonyms. In the excerpt given below, the Greek editor lists synonyms for ammonia/ammonium that recollect several key ideas at the heart of Greek alchemy. The text thus gives some of the oldest names for ammoniacal compounds, while illustrating the mind-set of the earliest known investigators.

The text translated here follows the Paris Grec 2327 manuscript, checked against Berthelot and Ruelle (1893, Vol II, p 338, Section V.2.4). It is untitled, simply starting "[Here] begins the following broad explanation [of] the work" (*archē tēs kata platos ton ergon exēgiseōs*) (f. 227 r.). The origins of the symbolism are uncertain and may be extremely ancient. Even in parts of India today, 'Milk of a Black Cow' is still spoken of as having magical properties. Round brackets (..) clarify, while square brackets [..] indicate missing or abbreviated text.

After distilling the first Water (dilute ammonium carbonate in water), the Water is redistilled up to seven times, including over lime, thereby concentrating aqueous ammonia; reference to Myrtle Leaves in the accompanying text appears to be a Jābirean synonym for the Water (al-Jildakī, Taslimi, 1954, p 532). Processing into ammonium is also indicated:

> ***onomatopoiia tou* [illegible] (*theiou*) *oxous kai* [illegible] (*hudatos*):** ***T**outo lege*[*tai*] *para t*[*on*] *philosoph*[*ōn*], [illegible] [illegible], [illegible] [illegible] (*oxos*), [illegible] (*magnēsia*) *leukē,* [illegible] *asbestou, ouron aphthoron, hudrarguros,* [illegible] *thalassēs, gala parthenou, onogala, kunogalon, gala boos melainēs,* [illegible] *stuptēri*[*as*], [illegible] *spodokrambēs, hudōr nitrou, kai dutikē, kai pnoē. Touto leukainē to sōma tēs* [illegible]*, ēg*[oun] *t*[on] *keikaumenon* ♀ (*chalkon*)*, touto pherei exō tēn phusin t*[*ēn*] *endon kekrumen*[*ēn*], *autē estin hē phusis hē nikōsa t*[*ēn*] *phusin, hē metalattousa tas phuseis, kai leiousa kai desmebousa* (*sic, desmeuousa*)*, hē egkuousa kai tiktousa, hē di' hou to pan apoteleit*[*ai*] (f. 227 v.).

> **Name-making of the divine vinegar and water: T**his is what they speak of among the philosophers, divine water, divine vinegar, white magnesia, water of lime, uncorrupt urine (or child urine), water-silver (i.e., Mercury), sea water, milk of a virgin, asses' milk, dog milk, milk of a black cow, water of alum, water of cabbage-ash, water of nitron, and western, and vapour/breath (also alluding to 'western breeze').
> [It is] this [that] whitens the body of magnesia, that is to say the burnt copper, this [that] brings out the nature hidden within (i.e., from within the burnt Copper – the black Magnesia in Silver-making and the Cinnabar in Gold-making), this is the Nature that triumphs over the Nature, it changes (or transmutes) the Natures, and smooths (or grinds/dissolves) and enchains, it conceives and gives-birth, through it the All is completed.

———
